# Antithrombotic and antibacterial surface coating based on spiky silver nanoparticles: A counterattack against clotting and biofilm

**DOI:** 10.1016/j.mtbio.2026.102762

**Published:** 2026-01-06

**Authors:** Cuong Hung Luu, Shehzahdi S. Moonshi, Akriti Nepal, Binura Perera, Dimple Sajin, Haotian Cha, Dieu Ngoc Nguyen, Nam-Trung Nguyen, Hang Thu Ta

**Affiliations:** a School of Environment and Science, Griffith University, Nathan, QLD, 4111, Australia; b Queensland Quantum and Advanced Technologies Research Institute, Griffith University, Nathan, QLD, 4111, Australia; c School of Engineering and Built Environment, Griffith University, Nathan, QLD, 4111, Australia; d Biomaterials and Nanotechnology Research Group, Faculty of Applied Sciences, Ton Duc Thang University, Ho Chi Minh City, Viet Nam

**Keywords:** Silver nanostar, Photothermal, PEG, Antithrombosis, Antibacterial, Thrombolysis, Antibiofilm

## Abstract

Blood-contacting medical devices such as vascular grafts, stents, and catheters are indispensable in life-saving interventions but remain prone to thrombosis and bacterial infection. These complications are often synergistic, with clot formation facilitating bacterial colonisation and biofilm growth, yet most surface coatings lack active countermeasures once thrombi or biofilms have developed. In this study, we hypothesised that integrating spiky silver-coated iron oxide nanoparticles (AgIONPs) with poly(ethylene glycol) (PEG) into a surface coating could provide both passive and active protection. AgIONPs offer strong photothermal properties under 808 nm laser irradiation for on-demand thrombolysis and biofilm disruption, while PEG contributes antifouling, anticoagulant, and biocompatible characteristics. The optimised AgIONPs–PEG coating exhibited safe photothermal heating (<45 °C), effectively lysed thrombi in static and dynamic models, and disrupted most biofilm biomass after a single irradiation cycle. Antithrombogenicity assays confirmed PEG's ability to reduce biofouling and improve haemocompatibility. Biocompatibility was further validated through *in vitro*, *in ovo*, and *in vivo* assays, with reduced immune-mediated inflammation. These findings highlight a multifunctional, responsive coating that could extend the lifespan and reliability of blood-contacting devices, offering a promising platform for next-generation photothermal materials in biomedical applications.

## Graphical abstract

**Figure:**
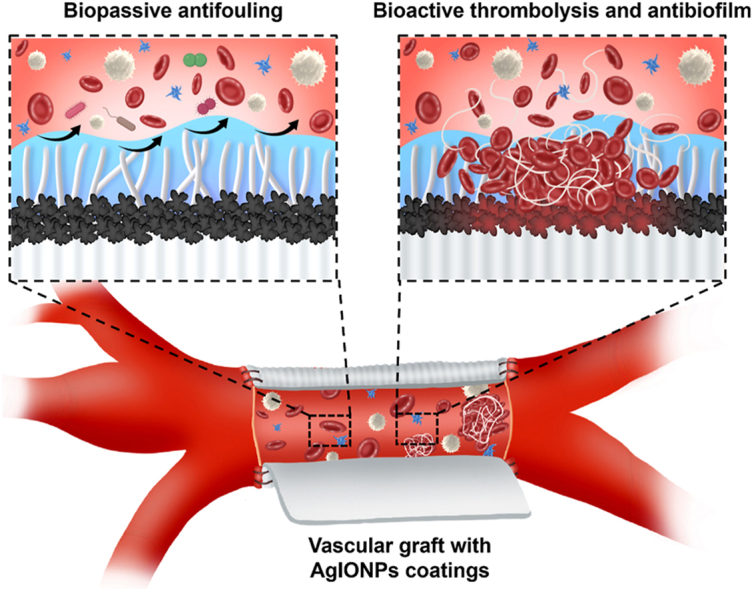

## 1 Introduction

Biomedical devices have become indispensable in modern healthcare, enabling the diagnosis, monitoring, and treatment of a wide range of diseases [1,2]. Among these, blood-contacting medical devices, such as vascular grafts, stents, catheters, haemodialysis membranes, and extracorporeal circulation systems, play a critical role in life-saving interventions [3]. However, when these devices come into direct contact with blood, they inevitably trigger a series of adverse biological responses [4,5]. The most prominent complications include surface-induced thrombosis and bacterial infection. Surface-induced thombosis results from the activation of coagulation cascades upon interaction between the device surface and blood components. Bacterial infection often arises from microbial adhesion and proliferation during implantation or prolonged use [6,7]. These phenomena are not only interdependent but also synergistic: thrombus formation can provide a scaffold for bacterial colonisation, while biofilm development can exacerbate inflammatory responses and further promote clot formation [8]. The consequences of these events are severe, ranging from device malfunction and loss of therapeutic efficacy to systemic infections, embolism, sepsis, and in extreme cases, mortality [8,9]. In addition, such complications often necessitate device removal or replacement, leading to additional surgical procedures, prolonged hospitalisation, and increased healthcare costs.

To mitigate these risks, surface coatings have been extensively explored as a means to counteract both thrombosis and bacterial colonisation [5,10]. By modifying the physicochemical and biochemical properties of the device interface, these coatings aim to improve haemocompatibility and reduce microbial adhesion. Numerous approaches have been reported in the literature, including the use of anticoagulant agents such as heparin, phosphorylcholine-based polymers, and hydrophilic coatings to resist protein adsorption, as well as antibacterial strategies based on antibiotics, silver coatings, or quaternary ammonium compounds [11,12]. While these strategies have achieved partial success, they also exhibit limitations. Many anticoagulant coatings function primarily through passive mechanisms, reducing initial protein adhesion and platelet activation but lacking the capacity to actively degrade or remove pre-existing thrombi. Similarly, most antibacterial coatings are effective in preventing bacterial adhesion at early stages but are far less capable of disrupting established biofilms, which are notoriously resistant to conventional antimicrobials [13]. Notably, there is a scarcity of multifunctional coatings that combine potent antithrombotic and antibacterial activity within a single, integrated system. This gap is particularly problematic in clinical scenarios, where clot formation or biofilm establishment has already occurred, because these passive coatings fail to halt progression, allowing the cascade of adverse effects to proceed unchecked.

Silver nanoparticles (AgNPs) have attracted considerable interest as an antimicrobial coating material due to their broad-spectrum bactericidal activity, which operates via multiple mechanisms including cell membrane disruption, interference with metabolic pathways, and induction of reactive oxygen species (ROS) [4,14]. However, despite their high antibacterial efficacy, AgNPs present significant drawbacks. The generation of ROS, while lethal to bacteria, can also provoke inflammatory responses in host tissue, induce cytotoxic effects, and paradoxically promote surface coagulation [15]. To address these limitations, AgNPs are often integrated with biocompatible, anti-inflammatory polymers such as heparin or poly(ethylene glycol) (PEG) [11,15]. In particular, PEG is valued for its “stealth effect” and its ability to reduce protein adsorption and opsonisation, thereby prolonging circulation time and minimising immune recognition. Nonetheless, even such conventional AgNP–polymer systems fall short when faced with pre-formed thrombi or mature biofilms, lacking the active mechanisms needed to remove these structures once they have developed.

To overcome these shortcomings, we propose the use of spiky silver-iron oxide nanoparticles (AgIONPs) synthesised by our group, which exhibit strong photothermal properties [[16], [17], [18], [19]]. The spiky morphology enhances localised light absorption, enabling efficient conversion of near-infrared (NIR) laser irradiation (808 nm) into heat. This photothermal effect can be harnessed for active thrombolysis and biofilm disruption: when exposed to NIR light, localised heating is sufficient to degrade fibrin networks and destabilise the extracellular matrix of biofilms, thereby restoring surface function and reducing microbial load. Importantly, the use of NIR light at 808 nm allows penetration through biological tissue to a certain depth to activate the photothermal effect in subcutaneous or intravascular devices [20]. When combined with PEG as a biocompatible matrix, AgIONPs can provide both passive and active protection: the PEG component offers haemocompatibility and reduced fouling, while the spiky AgIONPs supply robust antibacterial and thrombolytic activity on-demand.

In fact, photothermal therapy (PTT) has emerged as a highly promising antithrombotic strategy due to its precise spatiotemporal control, minimally invasive operation and favourable clinical safety profile [21]. Numerous studies have demonstrated the strong therapeutic potential of targeted PTT systems, typically relying on composite nanocarriers functionalised with specific targeting ligands. For instance, biomimetic platelet-camouflaged nanoparticles achieved thrombolysis efficiencies of up to 90 % in a femoral vein thrombosis model by combining PTT with rivoxaban release [22]. Likewise, metal–organic framework-derived carbon nanostructures, engineered for targeted delivery, successfully integrated PTT with photodynamic therapy and produced an 87.9 % reperfusion rate in rat models [23]. Building upon these concepts, our group has, for the first time, applied PTT within the context of surface coatings for blood-contacting materials and devices. This approach aims to provide an on-demand mechanism for addressing clot formation occur over time on the coated surface. This represents a novel translation of PTT principles into the domain of surface engineering for cardiovascular applications.

**Scheme 1:**
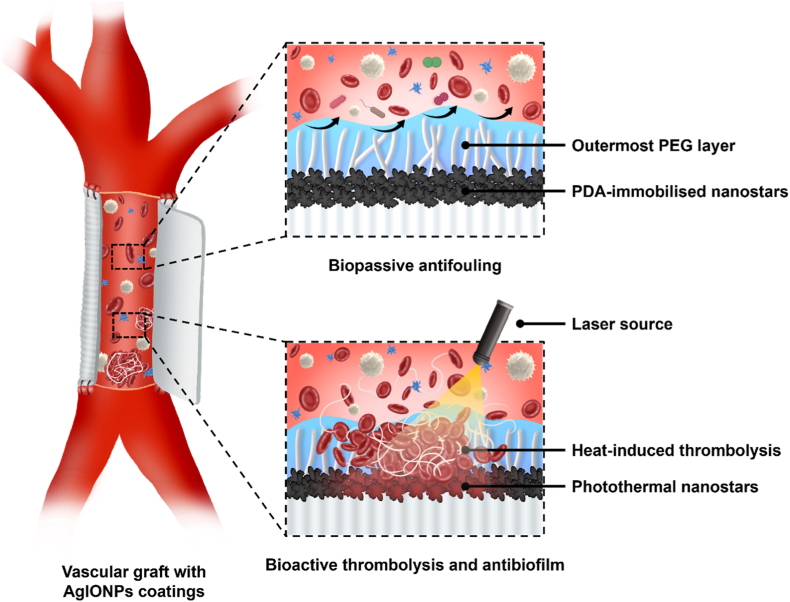
Illustration of AgIONPs and PEG-coated vascular graft for anticoagulant and antibacterial applications. The AgIONPs/nanostars layer is immobilised on the inner surface via PDA, providing bactericidal activity, while the outer PEG coating functions as an antibiofouling, antiplatelet, and passive anticoagulant barrier. In cases where thrombosis or biofilm develops on the surface, an 808 nm laser is employed to activate the photothermal properties of the silver nanostars, enabling thermal lysis of blood clots or disruption of biofilm.

In this study, we hypothesise that spiky AgIONPs with photothermal capability, integrated within a PEG-based surface coating, will deliver dual passive and active functions for antithrombosis and antibacterial protection under NIR laser exposure, as illustrated in Scheme 1. The surface coatings are thoroughly characterised using advanced analytical techniques to determine their morphology, roughness, surface energy, and chemical composition. Haemocompatibility and antibacterial performance are evaluated both in the absence and presence of laser activation to assess the potential for real-world application. Furthermore, biocompatibility is investigated from *in vitro* cell studies to *in vivo* animal models, enabling a comprehensive evaluation of the translational potential of this smart material platform. Collectively, our findings demonstrate a novel multifunctional coating strategy that addresses the persistent challenges of thrombus and biofilm management on blood-contacting medical devices, with implications for extending device lifespan, improving patient outcomes, and reducing the burden of device-associated complications.

## 2 Materials and methods

### 2.1 Materials

Ammonium iron(II) sulphate hexahydrate ((NH_4_)_2_Fe(SO_4_)_2_⸱6H_2_O, 99 %), iron(III) chloride (FeCl_3_, ≥99.99 %), silver nitrate (AgNO_3_, ≥99.0 %), dopamine hydrochloride (≥98 %), and poly(ethylene glycol) methyl ether thiol (PEG, *M*_n_ 2000 Da). Additional reagents were obtained from either Sigma-Aldrich (Missouri, United States) or Thermo Fisher Scientific Inc. (Massachusetts, United States). All chemicals were procured and utilised as received, without any further purification. The substrates used in this investigation were fabricated from pure poly(ethylene terephthalate) (PET) supplied by a local vendor. The PET sheets were cut into circular substrates measuring 6 mm in diameter and approximately 1 mm in thickness, thereby enabling their application in both *in vitro* and *in vivo* evaluations. Blood products were obtained from healthy volunteers with informed consents under the guidelines of human ethics approved by Griffith University human ethics committee (approval number: 2021/598).

### 2.2 Synthesis of iron oxide nanoparticles

Citrate-coated iron oxide nanoparticles (IONPs) were synthesised via a high-temperature co-precipitation method [16]. Initially, 2.7 g of ammonium iron(II) sulphate hexahydrate and 2.4 g of anhydrous iron(III) chloride were dissolved in 40 mL of deionised water within a three-necked flask, maintaining constant stirring at 500 rpm. Subsequently, 2 mL of citric acid (25 mg mL^−1^) was introduced, and the mixture was incubated in a 95 °C oil bath under an argon purge for 30 min. The formation of Fe_3_O_4_ nanoparticles was initiated by the slow injection of 10 mL of 30 % ammonia solution into the flask. After 1 h, the argon flow was halted, and the reaction mixture was cooled to ambient temperature before centrifugation at 4350×*g* for 6 min, after which the supernatant was discarded. The resulting precipitate was re-dispersed in 25 mL of citric acid buffer (20 mg mL^−1^, pH 5.2) using a probe sonicator, followed by centrifugation under the same conditions. This washing and re-dispersion process was repeated until the nanoparticles exhibited uniform dispersion. Finally, the citrated IONPs were dialysed in 10 kDa MWCO tubing against 5 L of DIW for two days, with water changes performed every 4–8 h.

### 2.3 Synthesis of spiky silver nanoparticles

The synthesis of silver-coated iron oxide nanoparticles (AgIONPs) was performed in accordance with a procedure previously established by our group [16]. An 8 mL suspension of iron oxide nanoparticles (IONPs) was prepared by adding 21.7 μL of 6.7 mg⸱mL^−1^ IONPs stock solution in a round-bottom flask containing DIW and maintained at 40 °C. Following this, 20 μL of 50 % hydroxylamine solution was added as a reducing agent, and the mixture was stirred. Subsequently, 70 μL of 11.4 mg mL^−1^ trisodium citrate solution was incorporated and thoroughly mixed. A 50 μL aliquot of 6.3 mmol L^−1^ AgNO_3_ solution was then added dropwise under vigorous stirring. After 10 min, a second 50 μL aliquot of AgNO_3_ was introduced, with this step repeated until a total of 250 μL had been added, during which progressive colour changes were observed. Thereafter, an additional 75 μL of citrate was introduced, and the denser AgIONPs were separated from the uncoated particles by centrifugation at 3500×*g* for 7 min. The supernatant was discarded, and the AgIONPs were re-dispersed in 200 μL of DIW, followed by dialysis against 5 L of DIW for two days. For PEG coating, a stock solution of PEG (2 mg mL^−1^) was prepared in DIW. The AgIONPs were re-suspended in 200 μL of DIW at a 1:1 wt ratio of AgIONPs to PEG and stirred at 450 rpm, which yielded PEG@AgIONPs. Any unbound PEG was subsequently removed by centrifugation at 1000×*g* for 6 min.

### 2.4 Preparation strategies for AgIONPs coatings

The immobilisation of AgIONPs onto the surface was achieved using a coating method employing a universal polydopamine coating, akin to methodologies detailed in prior studies [12]. While polydopamine can potentially immobilises AgIONPs, it has been noted to augment the adhesion of proteins, platelets, and red blood cells due to its reactive functional groups [5]. Given the exposure of the polydopamine network to the external environment, we sought to address these limitations by integrating PEG through three distinct preparation strategies. In the initial synthesis approach, PET surface was coated via immersion in a solution containing dopamine hydrochloride (2 mg mL^−1^) and AgIONPs in 100 mM Tris-HCl buffer at pH 8.5 for 2 h, obtaining PDA/AgIONPs coatings. In the subsequent method, PEG@AgIONPs were entrapped within PDA using a similar approach, resulting in PDA/PEG@AgIONPs where PDA is the outermost layer. This approach is still considered to potentially expose active functional groups, thereby increasing the likelihood of coagulation reactions occurring. To address this, we refined the original method by further modifying the PDA/AgIONPs surfaces with PEG, yielding PEG/PDA/AgIONPs where PEG is the outermost layer. Specifically, after forming the PDA/AgIONPs layer as described above, the samples were subsequently immersed in a PEG solution at a concentration of 0.5 mg mL^−1^, thereby yielding PEG/PDA/AgIONPs. In all strategies, the concentration of AgIONPs was varied at 0.1, 0.2, and 0.5 mg mL^−1^. To determine the optimal coating strategy, protein adsorption was selected as the representative evaluation parameter, as proteins are among the primary factors responsible for triggering surface-induced coagulation. The final optimised method (PEG/PDA/AgIONPs coatings) was subsequently employed throughout the study, with samples designated as Ag1, Ag2, and Ag5, corresponding to the concentrations of AgIONPs used for coating.

For assessments performed using a well plate, the optimised coating was applied directly to the base of each well following the same procedure as previously described. For the coating of the microfluidic device, the internal channel surface was treated following the optimised strategy, in which a solution containing 0.2 mg mL^−1^ AgIONPs and 2 mg mL^−1^ dopamine was introduced to fully fill the channel for 2 h. To visualise the channel, Cy5-amine was conjugated with carboxyl-terminated PEG (*M*_n_ 7500 Da) using an EDC-mediated coupling reaction at a molar ratio of 1.2:1:1.2 in aqueous solution at room temperature overnight. The channel was then coated with PEG-Cy5 via the remaining thiol group of PEG, after which it was thoroughly rinsed three times with PBS to remove unreacted reagents and immediately used.

### 2.5 Sterilisation of AgIONPs coatings

For the preparation of samples intended for both *in vitro* and *in vivo* experiments, sterilisation was carried out using 70 % alcohol. After sterilisation, the surfaces were left to dry under sterile conditions. The samples were then either immersed in phosphate-buffered saline (PBS) for 2 h prior to immediate use or rinsed with DIW, air-dried, and stored for later application.

### 2.6 Characterisation of AgIONPs coatings

**Morphology.** The surface morphology of the coatings was examined using scanning electron microscopy (SEM, Apreo 2S Scanning Electron Microscope, Thermo Fisher Scientific Inc., United States) and further characterised by atomic force microscopy (AFM, Park NX20, Park Systems Corporation, South Korea).

**Elemental analysis.** The Ag and Fe contents in the nanoparticle samples were quantified using inductively coupled plasma optical emission spectrometry (ICP-OES, Optima 8300DV, PerkinElmer Inc., United States). Elemental distribution mapping of the surface coatings was visualised by energy dispersive spectroscopy (EDS, Apreo 2S Scanning Electron Microscope, Thermo Fisher Scientific Inc., United States). Atomic composition and surface elemental content were further characterised by X-ray photoelectron spectroscopy (XPS, Kratos Axis Ultra XPS, Kratos Analytical Ltd., United Kingdom).

**Spectroscopy.** The chemical properties of the samples were examined using Fourier-transform infrared spectroscopy (FTIR, Spectrum Two, PerkinElmer Inc., United States). Ultraviolet–visible (UV–Vis) absorbance and fluorescence emission were subsequently measured with a microplate reader (CLARIOstar® Plus, BMG Labtech GmbH, Germany).

**Surface characteristics.** Furthermore, the thickness of the surface coatings on the PET films was determined using filmetric reflectometer, specifically a spectroscopic ellipsometer (Filmetrics F54, KLA Corporation, United States). Water contact angle (WCA) measurements for the coatings were performed at ambient temperature using the contact angle system (Attension® Theta Flex, Biolin Scientific AB, Sweden).

**Functionality density (AOII assay).** AOII was employed to quantify the amine groups present on the surface. The pH of the AOII solution was first adjusted to 4 using a hydrochloric acid buffer, after which the samples were immersed in 100 μL of this solution for 2 h. Following incubation, the samples were rinsed with hydrochloric acid at the same pH, and the surface-bound AOII was subsequently desorbed using a sodium hydroxide solution adjusted to pH 11. A 100-μL aliquot of the desorbed AOII solution was then analysed for absorbance at 485 nm using a microplate reader. The concentration of amine groups was calculated from the absorbance data by referencing a pre-established standard curve.

**Computational modelling.** COMSOL Multiphysics software (COMSOL AB, Sweden) was utilised to simulate the fluid dynamics within the microfluidic device, based on the actual dimensions employed in the experimental setup.

**Photothermal properties.** The photothermal properties of AgIONPs and the coated surfaces were assessed under irradiation with an 808 nm infrared laser using a custom-built stabilised infrared fibre laser system equipped with an adjustable intensity controller, allowing intensity variation from 1.0 to 2.0 W/cm^2^ and irradiation times ranging from 3 to 5 min. Temperature changes were recorded at defined time intervals, according to the experimental design, using a thermal camera (FLIR C5, Teledyne FLIR LLC, United States).

### 2.7 Cytocompatibility of AgIONPs coatings

**Cell culture.** Mouse endothelial cells (SVEC4-10, ATCC CRL-2181, passages 10) and mouse macrophages (RAW 264.7, ATCC TIB-71, passage 20) were cultured in low-glucose Dulbecco's Modified Eagle's Medium (DMEM, Thermo Fisher Scientific Inc., Massachusetts, USA) supplemented with 10 % fetal bovine serum and 1 % penicillin/streptomycin. All cultures were maintained in a humidified incubator at 37 °C under a controlled atmosphere containing 5 % CO_2_.

**ROS induction.** The production of ROS by macrophages in response to the surface coatings was quantified using the ROS probe 2′,7′-dichlorofluorescein diacetate (DCFDA, 50 μM). RAW 264.7 cells were seeded into a coated 96-well plate at a density of 1 × 10^4^ cells per well and cultured for 24 h. Bare well maintained in DMEM alone were used for comparison. After incubation under standard culture conditions, the medium was replaced with 100 μL of 50 μM DCFDA in PBS, followed by a 30-min incubation. Fluorescence intensity (FI) was then recorded at an excitation wavelength of 485 nm and an emission wavelength of 535 nm using microplate reader.

**Cell viability.** The cytocompatibility of the AgIONPs coatings was assessed using the LIVE/DEAD™ cell viability assay, employing SVEC4-10 endothelial cells (CRL-2181, passage 10) and RAW 264.7 macrophages (ATCC TIB-71, passage 21) as representative *in vitro* models. SVEC4-10 and RAW 264.7 cells were seeded into coated 96-well tissue culture plates at the density of and 1 × 10^4^ cells per well. Following a 24-h incubation under standard culture conditions, the LIVE/DEAD™ assay was conducted in accordance with the manufacturer's instructions. Fluorescence images were acquired using an inverted microscope (CKX53, Olympus Corporation, Japan) and cell viability was quantified based on the FI of calein AM measured at an excitation wavelength of 496 nm and an emission wavelength of 516 nm.

(1) $$\text{Cell}\;\text{viability}\;\left(\%\right)=\frac{{\text{FI}}_{\text{sample}}-{\text{FI}}_{\text{blank}}}{{\text{FI}}_{\text{control}}-{\text{FI}}_{\text{blank}}}\times 100\%$$

### 2.8 Haemolytic activity of AgIONPs coatings

For the haemocompatibility evaluation of silver-coated surfaces, whole blood was collected from healthy volunteers into vials containing 3.2 % sodium citrate with informed consents under the guidelines of human ethics approved by Griffith University human ethics committee (approval number: 2021/598). Each experiment was conducted in triplicate, employing PBS as the negative control and 1 % Triton X-100 as the positive control. Red blood cells (RBCs) were obtained by centrifuging the citrated whole blood at 1000×*g* for 15 min, followed by discarding the plasma fraction. The RBC pellet was subsequently washed twice with PBS and diluted at a ratio of 1:50 in PBS. A 400 μL aliquot of the diluted RBC suspension was then incubated with the surface coatings in an Eppendorf tube for 24 h at 37 °C under mild agitation at 200 rpm. After the incubation period, the samples were centrifuged again at 1000×*g* for 15 min, and the supernatant was carefully collected. The optical density (OD) of the supernatant was measured at 545 nm using a microplate reader, and the percentage of haemolysis was calculated according to the equation provided below.

(2) $$\text{Haemolysis}\;\left(\%\right)=\frac{{\text{OD}}_{\text{sample}}-{\text{OD}}_{\text{negative}}}{{\text{OD}}_{\text{positive}}-{\text{OD}}_{\text{negative}}}\times 100\%$$

### 2.9 Protein adsorption on AgIONPs coatings

The protein-repellent performance of AgIONPs coatings was evaluated using bovine serum albumin (BSA), fibrinogen (FBG), and platelet-poor plasma (PPP) as representative protein models. In this assay, the substrates were first immersed in PBS at 37 °C for 1 h to equilibrate and then incubated in 100 μL of separate protein solutions, 5 mg mL^−1^ BSA, 1 mg mL^−1^ FBG, and 10 % PPP, for 2 h in a 96-well plate, simulating the physiological protein levels. Following incubation, the samples were rinsed three times with PBS to remove loosely bound proteins, after which they were treated with 100 μL of 2 wt% sodium dodecyl sulphate solution and subjected to sonication for 30 min to detach adsorbed proteins from the surface. The amount of protein released from each sample was quantified using the bicinchoninic acid method with the Micro BCA™ Protein Assay Kit (Thermo Fisher Scientific Inc., Massachusetts, United States), following the manufacturer's protocol. Absorbance was recorded at 562 nm using a microplate reader, and all measurements were repeated in triplicate to ensure statistical robustness.

### 2.10 Platelet adhesion on AgIONPs coatings

Citrated whole human blood was initially centrifuged at 1500 rpm for 15 min to obtain platelet-rich plasma (PRP). A 100 μL aliquot of PRP was then dispensed onto the surfaces of the coated 96-well plate and incubated for 2 h at 37 °C. Following incubation, the samples were rinsed three times with PBS to remove non-adherent cells. The platelets attached to the surfaces were stained with DiOC6 at a concentration of 1 μg mL^−1^ in PBS for 30 min at ambient temperature, following by washing with PBS once [24]. Visualisation was performed using fluorescence microscopy. Quantification of platelet adhesion was carried out by enumerating platelet cells with ImageJ software (National Institutes of Health, USA), applying a gating threshold of 3 μm in diameter to ensure accurate identification and counting.

### 2.11 Antithrombogenicity of sPASP coatings

Activated partial thromboplastin time (APTT) and thrombin time (TT) assays were performed to assess the influence of AgIONPs-coated surfaces on the coagulation system. Fresh anticoagulated human blood was centrifuged at 3000 rpm for 15 min to obtain platelet-poor plasma (PPP). For the APTT assay, 50 μL of PPP was combined with 50 μL of APTT reagent (Dade® Actin FSL, Siemens Healthineers, Erlangen, Germany) and incubated at 37 °C for 30 min. The mixture was then applied to both uncoated and coated surfaces in glass tubes, after which 50 μL of 0.025 M calcium chloride was added, and the clotting time was recorded manually by tilting the tubes at 10-s intervals. For the TT assay, 100 μL of PPP was placed onto the test specimens in glass tubes, followed by the addition of 50 μL of TT reagent (Dade® Thrombin Reagent, 50 IU·mL^−1^, Siemens Healthineers, Erlangen, Germany) at 37 °C, and the clotting time was subsequently determined manually by tilting the tubes every 30 s.

### *2.12* *In vitro* thrombolysis of AgIONPs coatings

*In vitro* human thrombus was prepared using donated blood sourced from the Australian Red Cross, in full compliance with ethical requirements (2021/598) approved by the Human Ethics Committee of Griffith University, Australia. Specifically, whole blood was centrifuged at 1500 rpm for 15 min to separate RBCs and PRP. A thrombus-mimicking mixture was then formulated by combining 95 % fresh frozen plasma with 5 % RBCs, reflecting the typical composition of human arterial thrombus [16]. To initiate coagulation on the surface, 50 μL of this mixture was combined with 1.25 μL of 1 mol L^−1^ CaCl_2_ and gently dispensed onto the AgIONPs-coated surfaces, which had been pre-positioned in glass tubes. These tubes were sealed with parafilm to minimise water loss and subsequently incubated at 37 °C for 15 min to enable clot formation. An initial approach was undertaken to qualitatively assess thrombolytic activity by measuring the thrombus area following laser treatment under conditions of 1.5 W cm^−2^ intensity, 4 min exposure, and 1 cycle. The thrombus area was quantified using ImageJ, and the outcomes were compared against freshly formed clots prepared in PBS, which served as the control.

A subsequent comprehensive investigation was carried out to compare the variations in clot mass alongside the corresponding changes in temperature across the different coating samples. Each glass tube was weighed before and after clot formation, as well as following laser irradiation. The initial and final temperatures were monitored using a thermal camera. A series of surface coatings prepared using concentrations of 0.1, 0.2, and 0.5 mg mL^−1^ AgIONPs were employed. For comparative assessment, PBS was utilised as a negative control in the photothermal process, whereas PDA coating was also assessed owing to its established photon-to-heat conversion capability [25]. After clot formation, thrombus-coated samples were exposed to an infrared laser system operating at 808 nm, with a beam diameter of 1 cm to ensure complete surface and thrombus coverage. Laser power density was adjusted to 1.0, 1.5, and 2.0 W cm^−2^, while irradiation durations of 3, 4, and 5 min were systematically investigated. Upon completion of irradiation, the lysed thrombus fluid was carefully removed via pipetting, and the percentage weight reduction was calculated to determine the thrombolytic performance of the coatings. The percentage of clot mass loss following laser-induced thrombolysis was computed according to the equation presented below.

(3) $$\text{Thrombolysis}\;\left(\%\right)=\frac{m_{\text{post}-\text{irradiation}}-m_{\text{plain}\;\text{tube}}}{m_{\text{post}-\text{formation}}-m_{\text{plain}\;\text{tube}}}\times 100\%$$

### 2.13 Antithrombosis and thrombolysis of AgIONPs coatings under the flow

The anticoagulant and thrombolytic performance of the materials was directly verified using a microfluidic model to reflect the physiological relevance of blood-contacting medical devices [5,26]. In this study, a commonly adopted *in vitro* flow system was used to assess the haemocompatibility of the coated microfluidic device, which was fabricated by bonding a polydimethylsiloxane (PDMS) chip to a glass slide. The device consisted of a single-channel chip measuring 20 mm in length, 255 μm in width, and 110 μm in height. A shear rate of 1000 s^−1^, representing physiological conditions, was achieved by setting the syringe pump to a flow rate of 31 μL min^−1^. Subsequently, the chip was perfused with fresh citrated blood for 2 h. At 0, 0.5, 1, and 2 h, the perfusion was stopped, and clot formation was assessed by staining with DiOC6 at a concentration of 1 μg mL^−1^ in PBS for 30 min, followed by single wash, before observing under a fluorescence microscope. The FI of DiOC6 was quantified using ImageJ software across multiple positions within the channel. To ensure reproducibility, three separate channels were tested as replicates.

To assess the capacity for clot degradation in scenarios where thrombus formation occurred on the coated surface, a laser was applied to activate the underlying AgIONPs layer via its photothermal effect. Initially, blood clots were generated by prolonging the perfusion of blood through the coated microfluidic chip for 5 h, facilitated by the elevated shear rate arising from the intrinsic design of the device's channel. Thrombolysis was then initiated by irradiating the surface with a laser under the optimum setting previously determined under static conditions in Section 2.12 (4 min, 1.5 W cm^−2^, and 1 cycle). In the same manner as described earlier, the thrombus was visualised by staining with DiOC6 and imaging under a fluorescence microscope, both prior to and following laser exposure. Ultimately, the FI measurement was also carried out (before and after laser treatment) using the DiOC6 signal in order to determine the thrombolysis percentage.

### 2.14 Antibacterial efficacy of AgIONPs coatings

The antibacterial performance of the AgIONPs coatings was evaluated in accordance with ISO 22196:2011 standards, with only minor modifications to the protocol [27]. Two bacterial strains, gram-negative *Escherichia coli* (*E. coli*, ATCC 25922) and gram-positive *Staphylococcus aureus* (*S. aureus*, ATCC 6538), were selected as representative models for the antibacterial assessment. Both strains were pre-cultured and subsequently diluted in nutrient broth to obtain test cultures at approximately 5 × 10^5^ CFU mL^−1^. A volume of 100 μL of each bacterial suspension was then applied onto the coated surfaces of a 96-well plate and incubated overnight at 35 ± 1 °C under a relative humidity of no less than 90 %, with PET serving as the control sample. Following incubation, the suspensions were further diluted to 1/1000 for *E. coli* and 1/200 for *S. aureus*. From these diluted suspensions, 100-μL aliquots were spread evenly onto agar plates and incubated for an additional 24 h. The number of viable bacterial colonies was subsequently enumerated, and the antibacterial rate was calculated according to the formula described below.

(4) $$\text{Antibacterial}\;\text{rate}\;\left(\%\right)=\left(1-\frac{N_{\text{treatment}}}{N_{\text{control}}}\right)\times 100\%$$

### 2.15 Antibiofilm efficacy of AgIONPs coatings

The antibiofilm performance of the coatings was further examined in 96-well plates against both *E. coli* and *S. aureus*. Specifically, 100 μL of bacterial suspension at a concentration of 5 × 10^5^ CFU mL^−1^ in brain heart infusion broth was added to the coated wells. The bacteria were incubated at 35 ± 1 °C under a relative humidity of no less than 90 % for 48 h to allow biofilm formation. The broth was then carefully aspirated, and the biofilms were stained with 0.1 % crystal violet. For AgIONPs-containing samples, the biofilms were subjected to laser treatment under pre-optimised photothermal conditions (4 min, 1.5 W cm^−2^, and 1 cycle) before undergoing the same staining procedure. The wells were imaged using a Sapphire FL Biomolecular Imager (Azure Biosystems Inc., United States) at an excitation peak of approximately 592 nm and an emission peak of around 636 nm. Biofilm biomass, with and without laser exposure, was quantified by dissolving the crystal violet in 100 μL of absolute ethanol. The resulting solution was transferred to a separate well plate, and the OD was measured at 590 nm to minimise interference from the intrinsic absorbance of AgIONPs within the coatings.

### *2.16* *In ovo* biocompatibility of AgIONPs coatings

The chick embryo chorioallantoic membrane (CAM) assay was carried out to assess the biocompatibility of the surface coatings by monitoring embryonic development in the presence of the tested specimens. Fertilised chicken eggs were incubated under standard conditions at 36.5 °C with 65 % relative humidity. On the tenth day of embryogenesis, a small window (1.5 cm × 1.5 cm) was carefully created in the eggshell to expose the underlying CAM. Sterilised AgIONPs-coated samples (Ag2) were then gently placed onto the CAM surface, and 30 μL of PBS was applied directly to maintain hydration and support tissue viability. Following this, the opening was sealed with paraffin film, and the eggs were returned to the incubator for continued observation under controlled conditions.

### *2.17* *In vivo* inflammatory response of AgIONPs coatings

**Subcutaneous implantation.** The animal experiments were performed in compliance with the Guidelines for the Care and Use of Laboratory Animals of Griffith University (approval number ESC/03/24/AEC). Healthy adult female C57BL/6 mice, each weighing between 18 and 21 g, were selected for the implantation procedures. A subcutaneous pocket, approximately 0.5 cm in length, was surgically created on the dorsal side of each mouse. Into each pocket, a single PET disc either uncoated or coated, measuring 6 mm in diameter, was implanted and retained *in situ* for 7 days. The surgical incisions were then carefully closed with nylon sutures (ETHICON™ Surgical Technologies, Ethicon Inc., United States). At the end of the implantation period, the skin tissue in contact with the implanted material was excised and fixed in 4 % paraformaldehyde to enable subsequent histological assessment. Each treatment group comprised five mice (*n* = 5).

**Histological procedure.** After fixation, the specimens were embedded in cryomoulds containing Tissue-Tek O.C.T. Compound (Sakura Finetek Japan, Tokyo, Japan) and rapidly snap-frozen in liquid nitrogen. Longitudinal sections, each with a thickness of 6 μm, were prepared and stained with haematoxylin and eosin (H&E) for both morphological and morphometric assessment. Additionally, selected sections were mounted onto silanised slides for immunohistochemical (IHC) analysis, targeting alpha-smooth muscle actin (α-SMA, Cat. ab184675) and CD163 (Cat. ab313666) supplied by Abcam (Cambridge, United Kingdom). Both antibodies were applied at the same dilution ratio of 1:100 and incubated for 24 h.

**Capsule thickness measurement.** Bright-field microscopy at a magnification of 10 × was utilised to capture the specimens. The average capsule thickness for each group was then quantified using ImageJ, ensuring consistency in measurement across samples. These quantified values were subsequently compared with those obtained from untreated skin specimens collected from healthy mice, which served as the control group.

**Numerical density of leukocytes.** The estimation of inflammatory cell numbers was carried out within both the capsule region and the neighbouring tissue. Quantification was conducted on five H&E-stained sections, each assessed within a standardised field area of 0.31 mm^2^. In each field, inflammatory cell populations, including neutrophils, lymphocytes, plasma cells, and macrophages, were enumerated using ImageJ, with a diameter gating range set between 15 and 30 μm and a circularity range of 0.2–1.0. The resulting data were then subjected to statistical comparison with those from the control group [28].

**Inflammatory reaction score.** The inflammatory response within the capsule was assessed by considering both the quantity and spatial distribution of inflammatory cells. Each implant was assigned a score based on the following classification: 0 – absence of inflammatory infiltrate (less than 5 cells·mm^−2^); 1 – mild infiltrate (5–15 cells·mm^−2^); 2 – moderate infiltrate (15–25 cells·mm^−2^); and 3 – severe or intense infiltrate (more than 25 cells·mm^−2^). This evaluation was performed on five animals per group, after which the mean score for each group was calculated [28,29].

### 2.18 Statistical analysis

All experiments were carried out in triplicate or in a greater number of repetitions to enhance both the reliability and the reproducibility of the findings. The results were presented as mean values accompanied by their respective standard deviations. Statistical analyses, alongside graphical representations, were undertaken using GraphPad Prism (GraphPad Software, LLC, Massachusetts, United States). The threshold for statistical significance was determined according to *p*-values and was denoted as follows: ns (not significant) for *p* ≥ 0.05, *p* ∗ < 0.05, *p* ∗∗ < 0.01, *p* ∗∗∗ < 0.001, and *p* ∗∗∗∗ < 0.0001.

## 3 Results and discussion

### 3.1 Characterisation of AgIONPs

In this study, spiky silver nanoparticles were selected for coating instead of those synthesised via conventional methods [11]. This choice was based on their distinctive surface geometry, which markedly enhances localised surface plasmon resonance (LSPR) and thereby directly improves light-to-heat conversion [30]. The irregular morphology increases multiple light-scattering events and broadens the plasmon resonance band, facilitating stronger and more tunable NIR absorption, which is particularly advantageous for deep tissue penetration [31]. Consequently, this leads to an increased photothermal conversion efficiency. Specifically, in this work, AgIONPs were utilised not only for their antibacterial properties but also for their photothermal capability, which could effectively destroy blood clots and disrupt biofilms if they were to form during device usage.

**Fig. 1:**
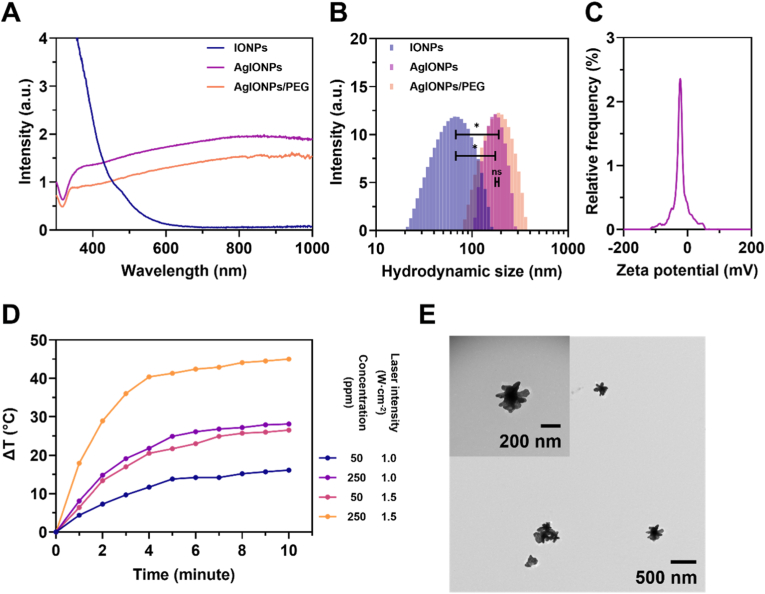
**Characterisation of synthesised nanoparticles. (A)** UV–Vis absorption spectra and **(B)** particle size distribution of IONPs, AgIONPs, and AgIONPs/PEG. **(C)** Zeta potential of AgIONPs. **(D)** Temperature elevation profiles based on the photothermal effect of AgIONPs in aqueous medium at varying concentrations and laser intensities. **(E)** TEM image with inset showing a magnified view of AgIONPs.

The synthesised nanoparticles were preliminarily characterised using UV–Vis spectroscopy. As shown in Fig. 1A, three nanoparticles including IONPs, AgIONPs, and AgIONPs/PEG, were compared based on their UV–Vis absorption spectra. The IONPs synthesised via the co-precipitation method exhibited no distinct absorption feature with a minor peak at 475 nm, indicating the absence of LSPR effect. In contrast, both AgIONPs and AgIONPs/PEG displayed a pronounced absorption maximum at approximately 800 nm, located in the NIR region. Besides, the calculated extinction coefficients of AgIONPs at different wavelengths in **Table S1** also pointed out that the value obtained at 808 nm turned out to be nearly the highest. This clearly confirmed the presence of LSPR following the silver coating of IONPs. Although PEG modification caused a slight reduction in absorption intensity, the spectral pattern remained unchanged, suggesting minimal influence on the LSPR effect. The absorption at around 800 nm was particularly advantageous for NIR laser applications, offering deep tissue penetration while enabling effective activation of the photothermal effect.

DLS results (Fig. 1B) revealed a notable increase in particle size from 69.1 ± 30.1 nm (PDI 18.9 %) for IONPs to 176.8 ± 39.7 nm (PDI 5.0 %) for AgIONPs, demonstrating a uniform particle size distribution after silver coating. PEG functionalisation led to a slight but non-significant size increase to 191 ± 64 nm (PDI 11.2 %). This size increment further confirmed the successful synthesis of AgIONPs, indicating the formation of a new coating layer over the original IONPs. In addition, the morphology of AgIONPs was observed to be spiky, as evidenced by the TEM image presented in Fig. 1E. The zeta potential of AgIONPs was determined to be −16.9 ± 0.9 mV (Fig. 1C), indicating a negatively charged surface, likely due to citrate adsorption. This negative surface charge not only contributed to nanoparticle stability by preventing agglomeration but also enhanced the interaction with dopamine moieties during surface immobilisation, thereby improving the overall efficiency of the AgIONPs coating strategy. The ICP-OES analysis results of AgIONPs are summarised in Table S2, showing an Ag content of 824.2 mg L^−1^ and an Fe content of 19.0 mg L^−1^. The calculated Ag:Fe mass ratio was approximately 43.3, whereas the initial reaction ratio was 1.2, indicating a substantial change in the ratio following the reaction.

For the photothermal effect, AgIONPs dispersed in DIW were evaluated under varying laser intensities (1.0 and 1.5 W cm^−2^) and nanoparticle concentrations (50 and 250 ppm). As shown in Fig. 1D, at a constant laser intensity, higher concentrations resulted in greater temperature increases. A similar trend was observed when varying the laser intensity, whereby at 1.5 W cm^−2^, the heating rate and maximum temperature rise were significantly higher than at 1.0 W cm^−2^. The maximum recorded temperature change within the tested range was approximately 45 °C above room temperature at 250 ppm and a laser intensity of 1.5 W cm^−2^. These findings demonstrated that the photothermal performance of AgIONPs was dependent on both nanoparticle concentration and the applied laser intensity. Moreover, the results suggested the need to optimise these parameters when applying coatings, in order to achieve a desirable temperature under laser irradiation, as temperatures exceeding 50 °C could potentially cause *in vivo* damage to healthy tissues [32]. In addition, the extinction coefficient of the nanostars was determined to be 2.23 × 10^12^ M^−1^ cm^−1^, while their photothermal conversion efficiency reached 26.2 %, as detailed in the **Supplementary Information** (**SI**) and Fig. S1.

The star-like morphology and efficient light-to-heat conversion of AgIONPs are closely linked to their synthesis process. When silver ions (Ag^+^) were introduced into a solution containing IONPs composed of ferrous (Fe^2+^) and ferric (Fe^3+^) ions, a galvanic replacement reaction occurred in which Ag^+^ was reduced to metallic silver (Ag^0^), while Fe^3+^ on the nanoparticle surface was oxidised. The resulting Ag^0^ domains acted as nucleation sites, enabling further reduction of Ag^+^ to metallic silver, which then extended into protrusions in a random manner. The use of silver as the outer metallic layer is particularly advantageous for enhancing the LSPR effect, owing to its high conductivity and abundant free electron density, which facilitates the efficient collective oscillation of conduction electrons upon light excitation. Moreover, these protrusions serve as sites of intense electromagnetic field concentration [33]. In addition, silver could be readily functionalised with thiol-containing organic groups, as the interaction between silver and sulphur is chemisorptive in nature, following the principles of soft acid–soft base interaction.

### 3.2 Surface characteristics of AgIONPs coatings

**Fig. 2:**
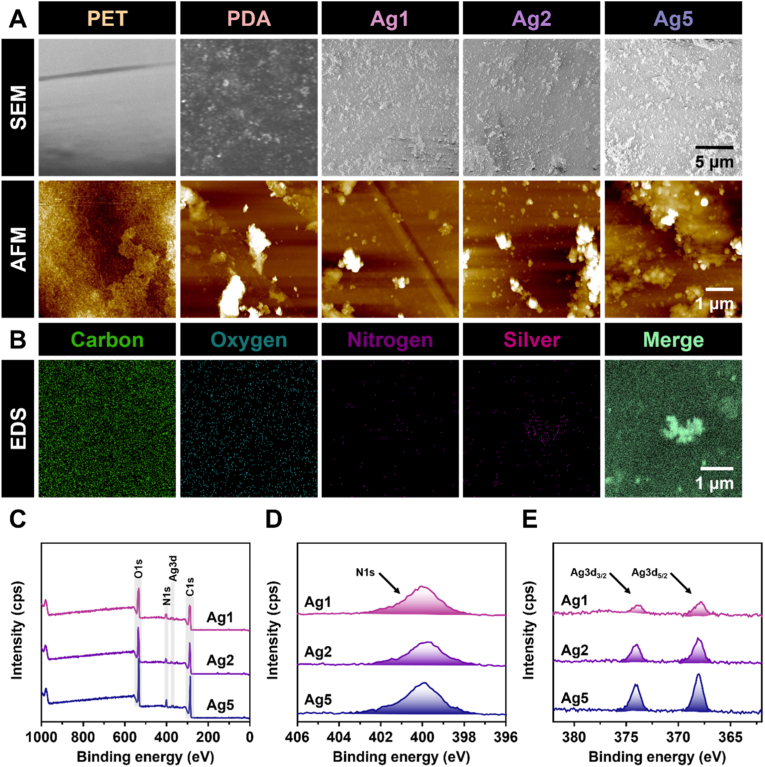
**Morphological and elemental analysis of surface coatings. (A)** SEM and AFM images of the bare surface and coated samples. **(B)** Representative EDS color mapping of elements C, O, N, Ag, and the merged image for the coating containing AgIONPs. **(C)** XPS survey spectra and high-resolution spectra of **(D)** N1s and **(E)** Ag3d for Ag1–5 samples. (For interpretation of the references to colour in this figure legend, the reader is referred to the Web version of this article.)

**Fig. 3:**
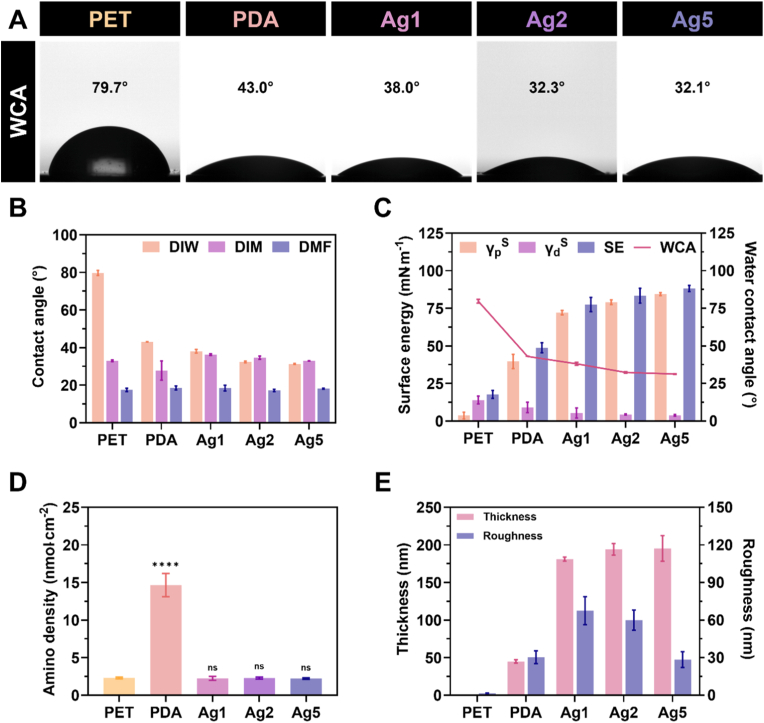
**Surface properties of the coatings. (A)** WCA captures and **(B)** contact angle measurements of the coatings using DIW, DIM, and DMF. **(C)** Surface energy and its components in comparison with WCA. **(D)** Amino group density determined by AOII assay. **(E)** Thickness and roughness of the coatings. Statistical comparisons of samples with PET were performed using one-way ANOVA test.

The surface properties were examined, as they can influence the anticoagulant performance of the material [5]. Initially, the surface morphology was visualised using SEM and AFM imaging, as shown in Fig. 2A. The SEM image of bare PET revealed a flat and smooth surface; however, following PDA coating, the surface exhibited a noticeable texture. This structural change was attributed to the deposition of PDA chains, which, during the reaction, could form nanoparticles that settled onto the PET surface, thereby increasing its roughness [34]. Surfaces containing AgIONPs (Ag1–5) displayed a similar texture but rougher. AFM imaging further confirmed the uniform topography of bare PET, whereas the PDA and AgIONPs-coated samples exhibited clear topographical irregularities, indicating a marked increase in roughness proportional to the AgIONPs concentration used for coating, which consistent with the SEM observations. Quantitative measurements of coating thickness obtained via ellipsometry and mean roughness derived from AFM analysis are presented in Fig. 3E. The coating thickness increased approximately fourfold after embedding AgIONPs, reaching around 190 nm, suggesting that silver was deposited as a monolayer. Although thickness showed a slight, non-significant rise among AgIONPs-coated samples, this trend suggested a higher PEG conjugation density with increasing AgIONPs content. Conversely, despite the general rise in roughness upon nanoparticle incorporation, a slight reduction in roughness was observed at higher AgIONPs densities, implying that a densely packed nanostar monolayer promoted a more uniform and consistent surface.

The FTIR spectra were examined to provide preliminary confirmation of the chemical functional groups present on the coating surfaces. As shown in Fig. S2, all spectra displayed a similar pattern to that of PET, with a characteristic trough at 723 cm^−1^ assigned to C–H bending. Following PDA coating, new troughs emerged at approximately 3340 and 2918 cm^−1^, corresponding to N–H and C–H/C=H stretching, respectively, indicating the introduction of a new material layer onto the PET surface. For surfaces bearing silver nanostars, the intensities of these two troughs increased noticeably. The broadening of peaks above 3000 cm^−1^ suggested an overlap with O–H signals, attributable to hydroxyl groups from citrate on the AgIONPs. The C–H/C=H stretching troughs were also intensified, implying the presence of ethyl groups from the PEG chains. Additionally, a peak at 1721 cm^−1^, characteristic of C=O stretching, and another at 1156 cm^−1^, attributed to aliphatic ether stretching (from PEG and PDA), were observed in the FTIR spectra of Ag1–5. Collectively, these spectral features confirmed the successful deposition of coating materials onto the bare PET surface.

For elemental analysis, EDS colour mapping was preliminarily employed. As shown in Fig. 2B, the elements C, O, N, and Ag were detected, with C and O being the most dominant species on the surface. In contrast, N, originating from the PDA layer, and Ag, present in the monolayer of AgIONPs, exhibited weaker signals, indicating that these elements were shielded beneath the outer PEG layer. In a separate analysis, amino group density determined using the AOII assay revealed that the PDA sample alone possessed a markedly higher surface amino concentration of 14.7 ± 1.3 nmol cm^−2^, as shown in Fig. 3D. In comparison, the remaining groups exhibited densities around 2.2 nmol cm^−2^, demonstrating the hindrance of these positively charged groups. Such reduced exposure of amino functionalities suggested a diminished propensity for protein, platelet, and other coagulation factor adhesion.

XPS analysis was carried out to determine the elemental composition and chemical states of the coatings. The wide‐scan spectra (Fig. 2C) displayed distinct peaks for O1s at 535.7 eV, N1s at 399.7 eV, C1s at 287.7 eV, and Ag3d at 374.2 and 368.2 eV. Peaks corresponding to O and C were the most prominent, in agreement with the presence of a PEG‐enriched outer layer that facilitated hydration, imparted anti‐biofouling properties, and reduced immune activation [35]. Consistent with the expected effects of PEG conjugation, the surface amine density showed a minimal existence around 6–7 % (Fig. 2D). As AgIONPs constituted the innermost layer, the Ag3d peak intensities were comparatively lower; however, Ag signals exhibited a gradual increase with higher AgIONPs loading (Fig. 2E). Quantitative data (Table 1) indicated Ag atomic percentages of 2.1 %, 2.5 %, and 3.3 % for Ag1, Ag2, and Ag5, respectively. The binding energies of Ag3d_5/2_ and Ag3d_3/2_ confirmed that silver was predominantly present in its metallic state Ag^0^ [36]. This trend observed for the coated AgIONPs was also consistent with the ICP‐OES results for Ag and Fe, as detailed in Table S2. Variations in other elemental signals were minimal, with C1s peaks at approximately 284.8 eV and 286.5 eV corresponding to C–C and C–O bonds characteristic of PEG chains.

**Table 1:** Elemental composition of surface coatings determined by XPS analysis.

Samples	C%	O%	N%	Ag%
**Ag1**	42.6	48.1	7.2	2.1
**Ag2**	42.1	49.3	6.1	2.5
**Ag5**	38.9	50.1	7.6	3.3

The surface energy of the coatings was subsequently investigated, as it reflects the hydrophilicity of the surface, which in turn governs its anti-fouling potential and capacity to minimise thrombus formation. As shown in Fig. 3A, WCA images indicated that PDA coating reduced the WCA by more than half, reaching 43.0°. Upon further incorporation of AgIONPs and PEG, the WCA continued to decline, with a decreasing trend proportional to the AgIONPs loading. Fig. 3B presents the measured WCAs and contact angles obtained using DIM and DMF as probe liquids. Based on these results, the surface energies of the coatings and their individual components were calculated using the method described in the SI. As shown in Fig. 3C, the surface energy of Ag1 increased markedly compared with PDA, from 48.8 ± 1.1 to 77.5 ± 1.6 mN m^−1^. Ag2 and Ag5 exhibited a gradual increase to 83.4 ± 1.6 and 88.2 ± 0.7 mN m^−1^, respectively. This trend suggested an enhancement in surface energy following AgIONPs incorporation, mainly attributed to an increase in the polar component and a reduction in the dispersive component. The results further confirmed the high hydrophilicity of the surface, which was strongly influenced by PEG. The presence of PEG facilitated the formation of a hydration layer, effectively reducing fouling and thus diminishing the likelihood of thrombus formation on the surface.

### *3.3* *In vitro* biocompatibility of AgIONPs coatings

**Fig. 4:**
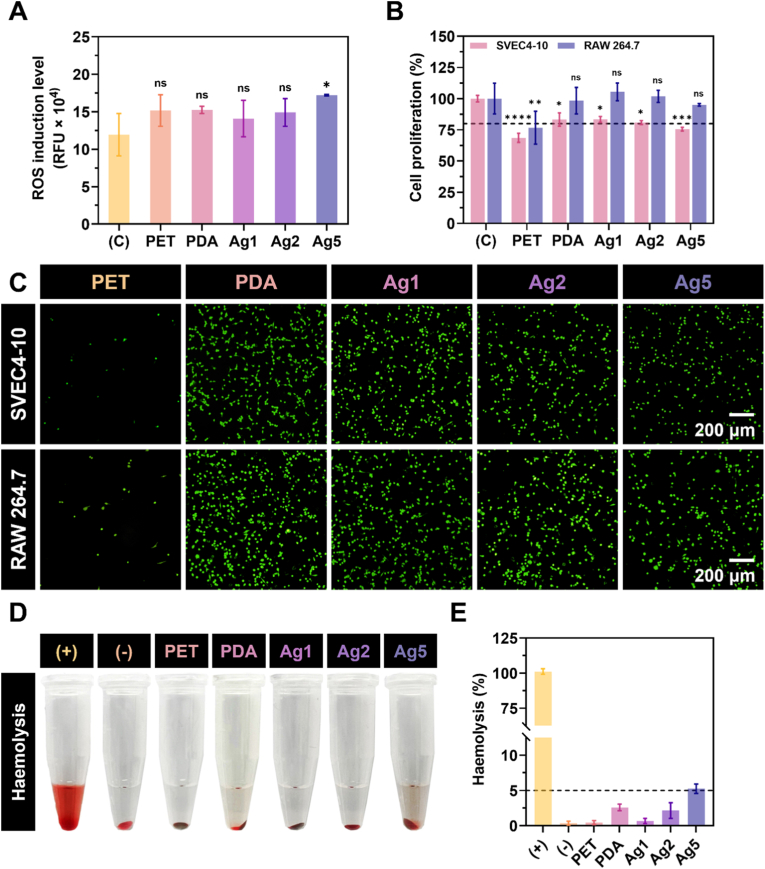
*In vitro* biocompatibility of surface coatings. (A) ROS generation levels of macrophages cultured on the surface coatings. **(B)** Proliferation of SVEC4-10 and RAW 264.7 cells on the surfaces and **(C)** representative images illustrating cell morphology using LIVE/DEAD™ reagents. **(D)** Images and **(E)** haemolytic activity of surface coatings. Statistical comparisons of samples with the control were performed using one-way and two-way ANOVA tests.

The biocompatibility of the surface coatings was first assessed by evaluating the level of ROS induced in macrophages. This analysis reflects the extent to which a material or treatment stimulates oxidative stress and, consequently, its potential to trigger inflammatory responses in immune cells. As shown in Fig. 4A, all tested samples produced slightly higher ROS levels in RAW 264.7 cells compared with the control, although the differences were minimal. Specifically, coatings with AgIONPs and PEG demonstrated a modest reduction in ROS generation relative to PET and PDA, with Ag1 and Ag2 recording values of 14.1 ± 2 and 14.9 ± 1.5 RFU × 10^4^, respectively. These findings suggest that PEGylation may enhance immune compatibility by reducing protein adsorption and immune cell recognition, thereby minimising inflammatory responses and prolonging material biocompatibility [37,38]. However, when the coating incorporated silver nanostars at a concentration of 0.5 mg mL^−1^, ROS levels generated by RAW 264.7 cells increased markedly, reaching 17.2 ± 0.1 RFU × 10^4^. This outcome is consistent with typical responses to silver-containing materials, as Ag^+^ release from AgIONPs can modulate immune activity by inducing oxidative stress through ROS generation, disrupting cell membranes, and altering signalling pathways [4]. Depending on the concentration and exposure duration, these effects may activate, suppress, or exert cytotoxic impacts on immune cells.

The biocompatibility of the coatings was further examined by assessing cell viability in both endothelial cells and macrophages. SVEC4‐10 and RAW 264.7 cell lines were selected owing to their physiological relevance in evaluating *in situ* interactions with antithrombotic material surfaces, as both are directly exposed to the coatings. Fig. 4B presents the percentage of viable cells cultured on each surface compared with the control, while Fig. 4C provides corresponding LIVE/DEAD™ staining images for visual confirmation. Overall, PET proved unsuitable for endothelial cell proliferation, with live cell density reaching only 68.6 ± 3 % compared to the control, despite not inducing cell death. This reduced cell proliferation on PET was largely attributable to cells failing to adhere to the surface and instead proliferating in the gap between the specimen and the well. For the other coated surfaces, SVEC4‐10 cell proliferation showed a gradual decline as AgIONPs content increased. While the coatings did not cause cytotoxicity, higher silver nanostar loadings impeded cell proliferation. Although most cells retained healthy morphology, Ag5 exhibited a cell proliferation of 75.6 ± 1.3 % compared to control, falling below the generally accepted threshold for biosafety [39]. In contrast, Ag1 and Ag2 maintained compatibility with endothelial cells, showing 83.4 ± 1.8 % and 80.9 ± 1.3 % proliferation, respectively. The ability of endothelial cells to grow robustly on the coatings also indicated the potential for long‐term applications through facilitation of endothelialisation. Macrophages demonstrated greater tolerance, yet their proliferation on PET was similarly suboptimal. As with endothelial cells, macrophage proliferation decreased progressively with higher AgIONPs densities, with Ag1 achieving the highest value at 105.5 ± 5.7 % and Ag3 the lowest at 95.0 ± 0.8 %, confirming strong compatibility with immune cells.

Given that red blood cells are also in direct contact with blood‐exposed materials, haemolytic activity was evaluated to assess potential cytotoxicity. Haemolysis testing is critical for determining whether a material disrupts erythrocyte membranes, which could compromise safety in biomedical use [40]. Fig. 4D and E respectively depict visual and quantitative results. As anticipated, PDA, with its high density of amino groups, destabilised red blood cell membranes, promoting haemolysis. A concentration‐dependent trend emerged, whereby increasing AgIONPs density correlated with greater haemolytic activity. Notably, Ag5 induced 5.3 ± 0.5 % haemolysis, a level considered potentially toxic for direct blood‐contacting applications.

### *3.4* *In vitro* antithrombogenicity of AgIONPs coatings

In evaluating the anticoagulant performance of the materials, protein adsorption was first examined, as proteins can compromise antithrombotic efficacy by promoting platelet adhesion and activation, thereby initiating the coagulation cascade and elevating thrombus formation risk [41]. Although discussed later in the work, the assessment of surface coating preparation strategies was conducted at an earlier stage to identify the most suitable approach. As shown in Fig. S3, results from the Micro BCA™ kit indicated high adsorption levels for all three model proteins on PET, PDA, PDA/AgIONPs, and PDA/PEG/AgIONPs surfaces. These findings supported the earlier hypothesis that PDA, while effective in anchoring AgIONPs, is unsuitable for plasma-contact applications, as it not only increases BSA adhesion but also significantly adsorbs FBG, a key coagulation factor and precursor of fibrin.

**Fig. 5:**
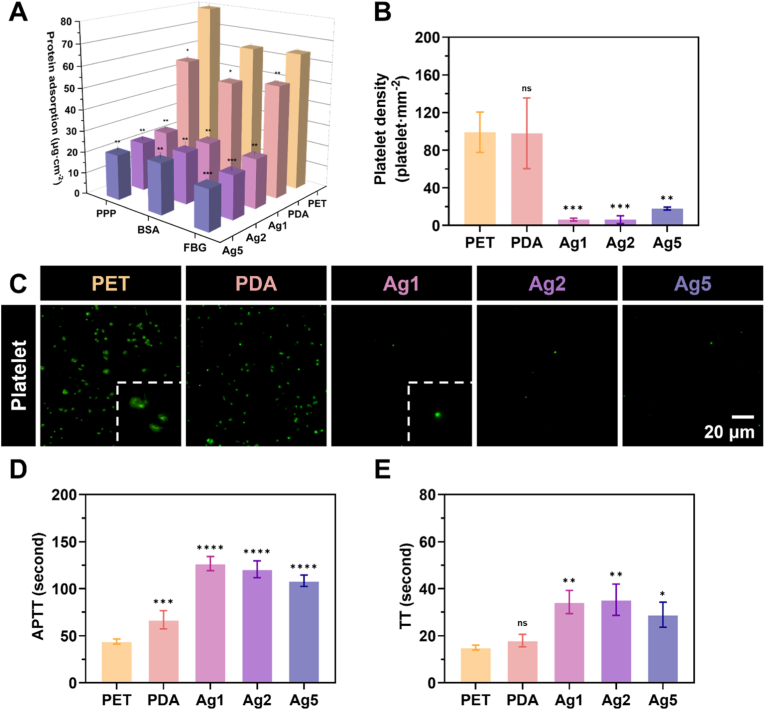
**Anticoagulant performance of surface coatings. (A)** Protein adsorption on the surfaces. **(B)** Platelet adhesion density quantified from **(C)** fluorescence images of DiOC6-stained platelets. Clotting times determined using **(D)** APTT and **(E)** TT assays. Statistical comparisons of samples with PET were performed using a one-way ANOVA test.

In contrast, PEG/PDA/AgIONPs coatings displayed marked antifouling capacity against all three model proteins. This outcome reinforced the rationale for adopting the PEG/PDA/AgIONPs synthesis strategy, denoted hereafter as Ag1, Ag2, and Ag5, corresponding to AgIONPs coating concentrations of 0.1, 0.2, and 0.5 mg mL^−1^, respectively. Protein attachment data for these optimised coatings, compared with PET and PDA, are presented as three-dimensional plots in Fig. 5A. For FBG, adsorption decreased progressively with higher AgIONPs content, from 23.1 ± 0.5 μg cm^−2^ for Ag1 to 19.1 ± 0.4 μg cm^−2^ for Ag5. A similar trend was observed for BSA and PPP, with substantially lower adsorption on PEG/PDA/AgIONPs surfaces compared to PET and PDA. This behaviour was attributed to the antifouling properties of PEG, where increased silver nanostar loading enhanced PEGylation and further reduced protein binding. These results aligned with previous surface energy analyses. In contrast, PDA, known for its strong adhesion to a broad range of substrates, not only served as a robust anchoring layer for AgIONPs but also exposed surface‐accessible catechol groups capable of binding proteins upon contact. This nature explained its comparatively higher protein adsorption profile.

Platelets were subsequently investigated as another key contributor to thrombosis [42]. Platelets promote thrombus formation by adhering to implant surfaces, undergoing activation, and aggregating into a platelet plug, which both initiates and amplifies the coagulation cascade [5]. This adhesion typically occurs through the recognition and binding of plasma proteins, such as fibrinogen or von Willebrand factor, already adsorbed onto the surface, facilitating receptor–ligand interactions that trigger platelet activation. In this study, platelets from PRP were used for evaluation. The quantitative and qualitative results are shown in Fig. 5B and C. PET exhibited the highest platelet adhesion among all samples, with 99.0 ± 17.5 platelets mm^−2^, closely followed by PDA at 97.9 ± 30.7 platelets mm^−2^. These results directly correlated with earlier protein adsorption data, confirming that greater protein adsorption led to increased platelet attachment. Moreover, platelets on PET and PDA displayed morphology spreading, indicative of activation and early signs of aggregation. In contrast, Ag1 and Ag2 surfaces witnessed very low platelet adhesion, averaging around 6 platelets mm^−2^. While Ag5 also showed significantly fewer adherent platelets than PET and PDA, its value rose to 18.0 ± 1.3 platelets mm^−2^, approximately double that of Ag1 and Ag2. Nevertheless, platelets on Ag-containing coatings did not exhibit activation features, highlighting the effectiveness of PEGylation in conferring antiplatelet properties.

To further assess anticoagulant potential, two standard coagulation time assays, APTT and TT, were performed. The APTT test measures the intrinsic and common coagulation pathways, detecting deficiencies or inhibitory effects on clotting factors such as VIII, IX, XI, and XII, while the TT test evaluates fibrinogen-to-fibrin conversion after thrombin addition, detecting abnormalities in fibrinogen content or function, or the presence of inhibitors such as heparin. The results are presented in Fig. 5D and E. All three AgIONPs-coated samples extended coagulation times. For APTT, Ag1 exhibited the longest clotting time at 126.7 ± 6.2 s, followed by Ag2 (120.7 ± 7.4 s) and Ag5 (108.3 ± 5 s). Conversely, TT results showed Ag2 with the highest value at 35.3 ± 5.4 s, followed by Ag1 at 34.3 ± 4 s, and Ag5 at 29.0 ± 4.3 s. Overall, these findings indicated that although PEGylated AgIONPs coatings possessed surface characteristics conducive to antifouling, at higher AgIONPs concentrations they might still compromise blood compatibility by promoting procoagulant effects [43].

### *3.5* *In vitro* thrombolysis of AgIONPs coatings

To initially assess the thrombolytic potential of the coatings, their photothermal performance was examined, with the results presented in Fig. S4. Overall, all three AgIONPs-containing coatings demonstrated effective heat generation, with the temperature rise increasing proportionally to the AgIONPs content on the surface. The maximum temperature elevations recorded were 9.7 °C for Ag1, 11.9 °C for Ag2, and 13.3 °C for Ag3. These coatings exhibited stable heating–cooling profiles compared with AgIONPs dispersed in DIW, which was attributed to the uniform and stable thermal conductivity of the solid PET substrate compared to liquid DIW solvent. Throughout the 60-min testing period, no signs of photothermal fatigue were observed, indicating that the temperature increase did not compromise the integrity of either the material or the coating. Notably, even the PDA-only coating displayed a measurable temperature variation under laser irradiation, although at a lower amplitude of approximately 4.4 °C, yet still maintaining stability and durability. This suggested that, in addition to AgIONPs, the immobilisation of silver nanostars via PDA enhanced the photothermal properties of the coating. Such behaviour of PDA was attributed to its conjugated π-electron system and broad light absorption within the visible–near-infrared range, enabling efficient conversion of absorbed photon energy into heat through non-radiative relaxation [44].

**Fig. 6:**
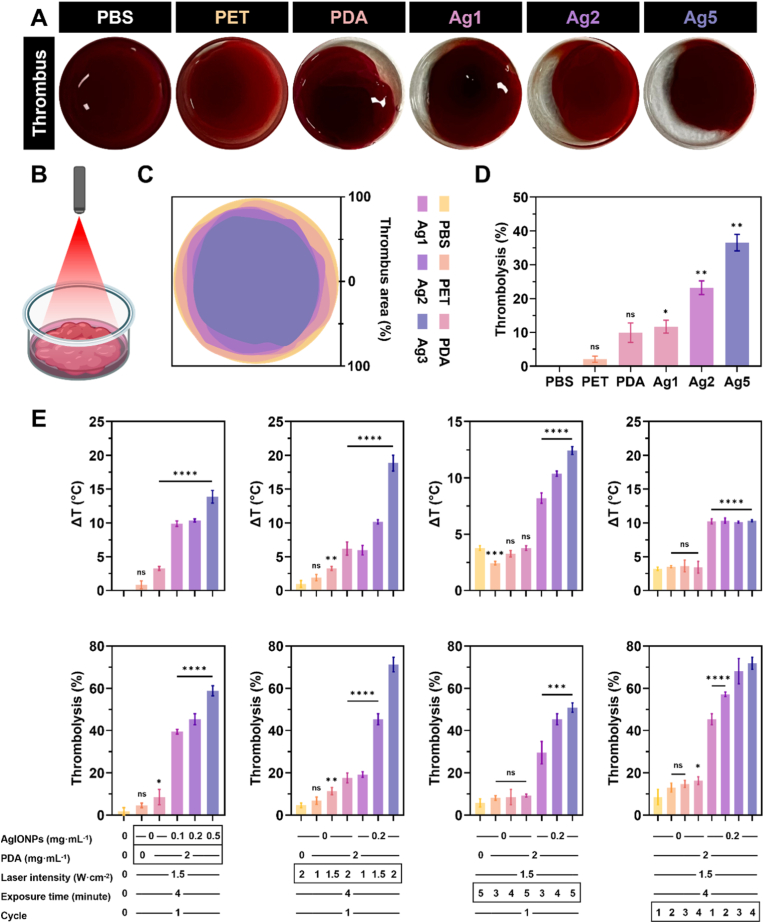
***In vitro* thrombolytic capability of surface coatings. (A)** Representative images of thrombus degradation following **(B)** laser irradiation at 1.5 W cm^−2^ for 4 min illustrated in BioRender. Luu, C. (2025) https://BioRender.com/diuncte. **(C)** Stacked thrombus area of the surfaces after thrombolysis and **(D)** thrombolysis measurements derived from thrombus area compared to untreated samples. Evaluation of changes in **(E)** temperature and **(F)** thrombolysis with variation of AgIONP coating content, laser intensity, exposure time, and treatment cycles. Statistical comparisons between samples and untreated controls were conducted using a one-way ANOVA test.

To evaluate the thrombolytic capacity of the coatings, thrombi were first formed on the surface and subsequently treated with laser irradiation to compare clot area reduction. The experimental setup was illustrated in Fig. 6B. In this setup, thrombi generated on the surface fully covered the base of the glass tubes, as shown for the PBS control in Fig. 6A. After irradiation at an intensity of 1.5 W cm^−2^ for 4 min, the samples were imaged, and clot areas were measured as shown and stacked in Fig. 6C, with area-based thrombolysis values calculated and presented in Fig. 6D. The PET sample exhibited virtually no change in clot area, whereas the PDA coating, despite producing only a modest temperature increase, reduced thrombus coverage by approximately 9.9 ± 2.8 %, closely matching the 11.7 ± 4.4 % reduction observed for Ag1, indicating an initial measurable effect. As anticipated, Ag2 and Ag5 demonstrated significantly greater thrombolysis, achieving reductions of 23.2 ± 11.0 % and 36.6 ± 16.7 %, respectively. This preliminary study suggested that heat generated by the surface coatings could shrink and fragment the clot, facilitating its removal using a pipette.

To provide a more comprehensive analysis, thrombolysis efficiency was further assessed based on thrombus mass, alongside photothermal performance. Variables such as the presence of PDA, AgIONPs concentration, laser intensity, exposure time, and irradiation cycle were systematically examined, as shown in Fig. 6E. Initially, the PDA-coated sample was evaluated, revealing that in the absence of AgIONPs, the temperature increased modestly by 2.6 ± 0.3 °C, resulting in 8.6 ± 2.9 % thrombolysis. In comparison, the uncoated control without PDA showed only a 0.6 ± 0.4 °C increase and 4.6 ± 0.9 % thrombolysis. Photothermal activity and thrombolytic efficiency in this group increased proportionally with laser intensity. Coatings containing silver nanostars exhibited a similar dose- and intensity-dependent behaviour, consistent with earlier findings. Among these, Ag5 produced the highest thrombolysis (71.2 ± 2.8 %) under 2 W cm^−2^ irradiation for 4 min, although it also generated a temperature rise of 18.9 ± 1.2 °C. Such a thermal increase, when added to the baseline of 37 °C, exceeded 50 °C, potentially inducing irreversible biochemical alterations such as protein denaturation and coagulation onset [45]. Conversely, Ag2 demonstrated milder, more clinically acceptable photothermal behaviour, with an 10.2 ± 0.3 °C rise and 45.3 ± 2.1 % thrombolysis under identical conditions.

An exposure time-dependent trend was also observed. For example, Ag2 irradiated at 1.5 W cm^−2^ for 5 min exhibited a temperature rise of 12.4 ± 0.4 °C and 50.8 ± 1.8 % thrombolysis. Comparing the two experimental parameters indicated that laser intensity had a stronger influence on photothermal variation. Under 1.5 W cm^−2^, extending exposure by 1 min increased the temperature by only 1.6 °C, whereas increasing intensity by just 0.5 W cm^−2^ at a fixed duration of 4 min led to a 6.8 °C rise. This highlighted the need for careful optimisation of laser power to ensure patient safety [46].

Furthermore, increasing the number of irradiation cycles produced negligible changes in peak temperature, which remained around 10.2 °C. This stability was attributed to the off-cycle allowing thermal equilibration, consistent with results in Fig. S4. However, thrombolysis improved with multiple cycles, reaching 71.8 ± 2.3 % without requiring excessive laser power, suggesting a practical and safer therapeutic protocol. Based on these findings, the optimal *in vitro* static thrombolysis parameters were identified as the Ag2 coating (0.2 mg mL^−1^ AgIONPs) with a laser intensity of 1.5 W cm^−2^, an exposure time of 4 min, and 3 irradiation cycles. Nonetheless, it should be noted that these optimised conditions were specific to the current *in vitro* static model and may require adjustment for other experimental or clinical settings.

### 3.6 Antithrombosis and thrombolysis of AgIONPs coatings under the flow

**Fig. 7:**
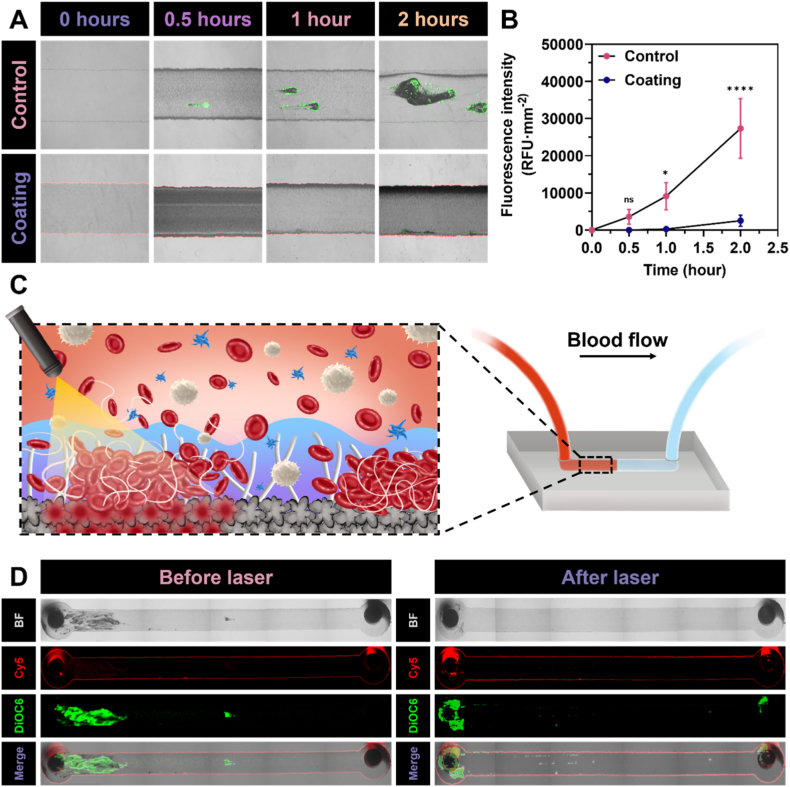
*In vitro* antithrombosis performance under flow conditions. **(A)** Merged images visualising the anticoagulant capability of the coated surfaces compared to uncoated surfaces and **(B)** DiOC6 fluorescence intensity measured over time. Bright-field images were merged with Cy5-labelled coatings (red) and DiOC6-stained platelets (green) to enable simultaneous visualisation of the surface layer and platelet adhesion. Statistical analyses were performed between data points at the same time interval for the two groups using a *t*-test. **(C)** Schematic representation of the thrombolysis assay conducted on a microfluidic device. **(D)** Images showing thrombus formation at the inlet of the microfluidic device under flow for 5 h, captured before and after laser irradiation for thrombus degradation. (For interpretation of the references to colour in this figure legend, the reader is referred to the Web version of this article.)

Shear rate played a crucial role in modulating surface-induced thrombosis by influencing platelet activation, adhesion, and aggregation on biomaterial surfaces [47]. At high shear rates, platelet activation and recruitment were accelerated via von Willebrand factor-mediated binding, thereby promoting rapid thrombus growth, whereas low shear rates favoured fibrin formation and the development of stable clots [47,48]. Therefore, assessing the thrombogenicity of surface coatings under flow conditions was considered essential. In this study, a single-channel microfluidic device model was employed to investigate thrombus formation under controlled shear rate conditions, using the optimised Ag2 coating and comparing it with an unmodified bare microfluidic device as the control. This configuration of this model enabled direct visualisation and monitoring of clot formation on the channel surface via microscopy, as illustrated in Fig. 7A.

Cy5-conjugated PEG was applied as the coating material, due to the difference in fluorescence intensity between the channel walls and its centre, the Cy5 red signal appeared exclusively along the channel walls. Platelets were stained with DiOC6 to allow clear *in situ* visualisation of clotting, shown as a green signal. In the control sample, thrombus formation began within the first 30 min, with platelet aggregation progressively intensifying clotting over time. After 2 h, a larger blood clot had formed, accompanied by noticeable changes in flow patterns. By contrast, in the Ag2-coated device, almost no platelet adhesion was observed during the first 30 min. Between 1 and 2 h, only a few small thrombi were detected along the channel walls, likely attributable to minor surface irregularities at the PDMS–glass interface introduced during oxygen plasma bonding, which could locally increase shear rate or provide anchoring points for platelets, thereby producing a localised DiOC6 signal. Overall, these signals were minimal compared with the pronounced clot growth in the control, as shown in Fig. 7B. The findings indicated that the Ag2 coating reduced thrombosis, which was attributed to the antifouling effect of the outer PEG layer, forming a hydration barrier that effectively suppressed coagulation.

Similar to the static thrombolysis model performed in glass tubes, the microfluidic system was also employed to investigate thrombolytic activity under flow conditions. The experimental setup and the simulation of laser application for thrombolysis under flow are illustrated in Fig. 7C. As in the previous assays, a shear rate of 1000 s^−1^ was applied for 5 h to promote blood clot formation. In this configuration, clots rarely developed in the middle of the channel but predominantly formed at the inlet, where they were subsequently enlarged following the growth of preformed clots. This observation confirmed the coating's strong resistance to thrombus formation on the channel surface. However, the unusual thrombus development at the inlet was further examined using COMSOL Multiphysics to simulate fluid dynamics, as illustrated in Fig. S5. The results revealed a localised increase in shear rate at the inlet, highlighted by warm-coloured regions in both Fig. S5A and S5B, caused by mechanical constraints in the microfluidic chip design. Such shear rate elevations could trigger platelet activation and initiate downstream coagulation cascades, thereby intensifying thrombosis from this location. Consequently, these inlet-associated clots were not considered indicative of reduced anticoagulant performance of the AgIONPs coatings.

Following clot formation, the entire clot-occupied area within the channel was irradiated under the optimised conditions (1.5 W cm^−2^, 4 min) but limited to a single cycle, after which flow was resumed. Although the optimisation experiments previously determined threee cycles as the most effective treatment, this single-cycle trial already achieved a substantial thrombolysis rate of 77.9 ± 10.7 %, serving as a proof of concept for thrombolysis under flow. The results further implied that enhanced therapeutic outcomes could be attained by increasing the number of cycles, laser intensity, or exposure duration, depending on application-specific parameters such as implant depth, clot volume, and treatment accessibility. Overall, the study successfully demonstrated that effective clot lysis could be achieved under shear rate influence with only a single irradiation cycle, highlighting the potential of this approach to counteract thrombosis forming on blood-contacting device surfaces *in situ*.

### 3.7 Antibacterial and antibiofilm of AgIONPs coatings

**Fig. 8:**
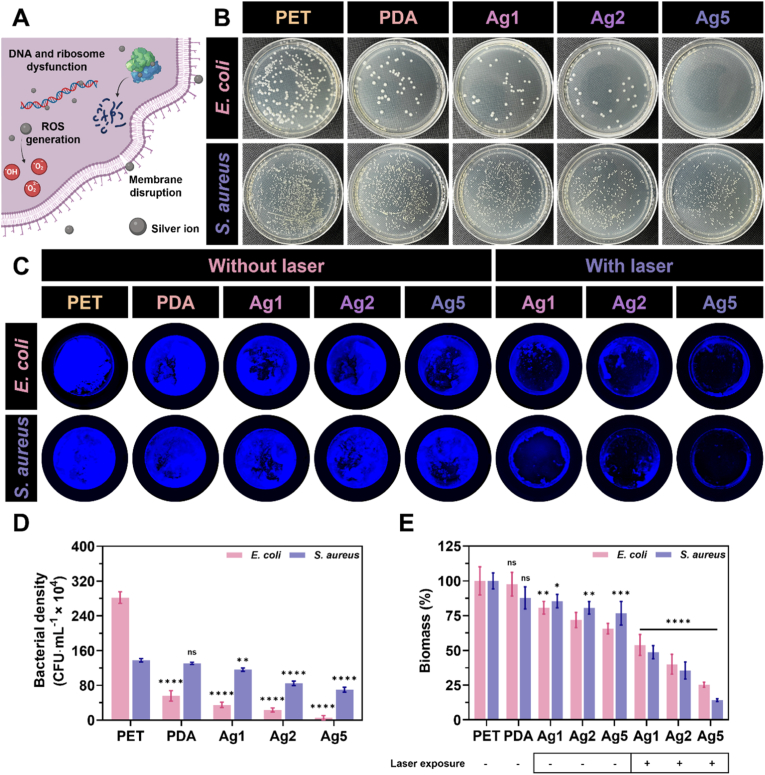
**Antibacterial performance of surface coatings. (A)** Proposed antibacterial mechanism induced by the release of silver ions from AgIONPs. **(B)** Colony forming units of *E. coli* and *S. aureus* following exposure to the surface coatings. **(C)** Inhibition of biofilm formation on the coatings and disruption of established biofilms through laser exposure. **(D)** Bacterial density after *in vitro* culture with the surface coatings. **(E)** Biofilm biomass stained by crystal violet and quantified before and after laser application to the coatings. Statistical comparisons were conducted between PET as a control and the other surfaces using one-way and two-way ANOVA tests. (For interpretation of the references to colour in this figure legend, the reader is referred to the Web version of this article.)

For the antibacterial evaluation of the AgIONPs coatings, two bacterial strains were selected to represent Gram-negative and Gram-positive species, namely *E. coli* and *S. aureus*. The antibacterial functionality of the surface coatings was hypothesised to result from the release of silver ions from the surface of the silver nanostars, which can act against bacteria through multiple mechanisms, including membrane disruption, ROS generation, and interference with DNA and ribosomal functions, as illustrated in Fig. 8A. To verify their bactericidal performance, both bacterial strains were cultured with the coated surfaces in nutrient broth, followed by colony counting. The qualitative and quantitative results are presented in Fig. 8B and D, respectively.

Overall, *E. coli* displayed lower tolerance than *S. aureus* when exposed to the AgIONPs coatings. PDA alone demonstrated a notable inhibitory effect against *E. coli*, consistent with previous reports, with bacterial counts reduced to 55.7 ± 9.8 CFU mL^−1^ × 10^4^ compared with 282.0 ± 11.0 CFU mL^−1^ × 10^4^ for the PET control [49]. As anticipated, the bactericidal effect against *E. coli* increased proportionally with the silver nanostar loading, with bacterial density declining from 35.0 ± 4.9 CFU mL^−1^ × 10^4^ for Ag1 to just 5.3 ± 4.1 CFU mL^−1^ × 10^4^ for Ag5. By contrast, although *S. aureus* followed a similar trend, this Gram-positive strain exhibited greater resilience than the Gram-negative counterpart. PDA coatings showed no significant difference from PET in their antibacterial activity against *S. aureus*, with a marked reduction only observed from Ag1 (116.4 ± 3.1 CFU mL^−1^ × 10^4^) to Ag5 (69.9 ± 4.6 CFU mL^−1^ × 10^4^). These findings suggested that PDA may carry positively charged amino groups or expose reactive catechol and quinone groups on the surface, which can generate ROS and compromise bacterial membrane integrity. Similarly, silver ions released from the AgIONPs likely synergised with the PDA layer to enhance bactericidal performance, thereby contributing to the overall antibacterial activity of the AgIONPs coatings [14].

The antibiofilm performance of the AgIONPs coatings was assessed by culturing bacteria in nutrient-rich medium. As shown visually in Fig. 8C and quantitatively in Fig. 8E, the AgIONPs-containing coatings exhibited substantial biofilm inhibition, although biofilm formation was still observed after 48 h of incubation. Among the tested samples, Ag5 demonstrated the most effective antibiofilm activity, reducing *E. coli* biomass to 65.6 ± 3.1 % and *S. aureus* biomass to 76.7 ± 6.9 % relative to PET controls. A concentration-dependent effect was evident, with higher AgIONPs loading corresponding to lower biomass levels. Consistent with earlier observations, *E. coli* displayed lower resilience to the silver nanostar-induced effects than *S. aureus*.

As hypothesised, laser irradiation was applied to disrupt the bacterial biofilm structure. When performed under the optimised photothermal conditions (1.5 W cm^−2^ and 4 min) but with only one irradiation cycle, the coatings still achieved a notable effect. Specifically, Ag1 retained 53.9 ± 6.1 % *E. coli* biomass and 48.7 ± 3.9 % *S. aureus* biomass, whereas Ag5 exhibited markedly stronger activity, leaving only 25.2 ± 1.6 % *E. coli* biomass and 14.2 ± 0.8 % *S. aureus* biomass. This experiment was sufficient to validate the proof of concept for biofilm disruption via the photothermal effect of AgIONPs coatings after 48 h of biofilm development in brain heart infusion broth. The results highlighted the potential of this approach not only for lysing blood clots but also for preventing biofilm formation on device surfaces, thereby mitigating the risk of device-associated systemic infections.

### *3.8* *In ovo* and *in vivo* biocompatibility of AgIONPs coatings

To further assess the biocompatibility of the AgIONPs coatings, an *in ovo* model closely approximating *in vivo* conditions was first employed using the CAM assay with chick embryos. In this experiment, the Ag2 coating, previously identified as the optimal formulation from earlier investigations, was implanted onto the CAM at day 11 of embryonic development. In a similar manner, PET together with saline addition was used as the control. The results, recorded in Fig. S6, display images from days 11, 12, and 13. By day 12, the CAM had begun to integrate with the implant, with both major and minor blood vessels developing normally and without signs of inflammation, indicating that the implant had almost seamlessly merged with the CAM. By day 13, the embryo exhibited typical turning and positioning prior to hatching behaviour, confirming healthy development without any adverse impact from the implant [50]. The PET surfaces also exhibited comparable outcomes, supporting the stable and healthy development of the embryos. Nevertheless, when compared with the samples incubated with saline, it became evident that the Ag2 samples more closely approached in promoting robust vascular development. These findings demonstrated the benign nature and compatibility of the material in an *in ovo* setting.

**Fig. 9:**
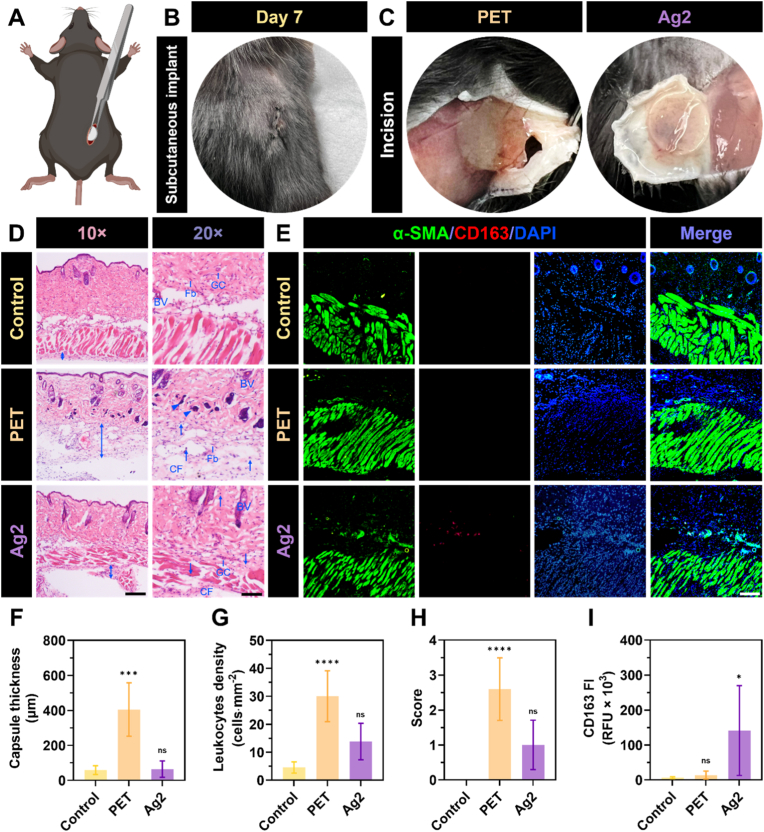
**Evaluation of inflammatory responses to subcutaneously implanted surface coatings in mice. (A)** Schematic representation of subcutaneous implantation in the C57BL/6 mouse model, created in BioRender. Luu, C. (2025) https://BioRender.com/dcsoumi. **(B)** Appearance of the suture site at the implantation region after 7 days. **(C)** Images of explanted plain PET and AgIONPs-coated specimens following surgical retrieval. **(D)** Representative H&E-stained tissue sections captured at 10 × and 20 × magnifications, with scale bars corresponding to 200 μm and 100 μm, respectively. Key histological observations included capsule layers (two-way arrows), inflammatory cells (one-way arrows), necrotic cells (triangular pointers), fibroblasts (Fb), multinucleated giant cells (GC), collagen fibres (CF), and blood vessels (BV). **(E)** Immunohistochemical staining using α-SMA (green) and CD163 (blue) antibodies, counterstained with DAPI. Quantitative analyses of **(F)** capsule thickness, **(G)** leukocyte density, **(H)** inflammation scores, and **(I)** CD163 FI were derived from the stained tissue samples. Statistical comparisons were conducted between healthy skin as a control and treated skin using one-way ANOVA tests. (For interpretation of the references to colour in this figure legend, the reader is referred to the Web version of this article.)

Subsequently, *in vivo* evaluation of inflammatory responses was performed in a C57BL/6 mouse model. Fig. 9A illustrates the implantation approach for the surface coatings, with experimental details described in Section 2.17. The wound closure site, photographed on day 7 prior to implant retrieval (Fig. 9B), appeared stable with no signs of infection, suggesting that the suturing procedure had no confounding influence on inflammation assessment. Upon retrieval (Fig. 9C), skin samples in contact with PET displayed a noticeably thicker fibrous encapsulation than those with the Ag2 coating. This encapsulation is a typical immune response to a foreign body, involving activation of immune cells such as macrophages, recruitment of fibroblasts, and subsequent collagen deposition to form a fibrous capsule, which in poorly biocompatible materials serves to isolate the implant, often leading to failure [51].

Microscopic analysis (Fig. 9D) and capsule thickness measurements (Fig. 9F) confirmed that healthy skin (control) and Ag2 coating implants exhibited significantly thinner capsules compared to bare PET, indicating superior biocompatibility of the coating. Leukocyte density measurements (Fig. 9H) from 20 × microscopic images showed a modest increase in infiltration for Ag2 coating implants (13.8 ± 5.8 cells mm^−2^) compared to control, but this was not statistically significant, whereas PET implants demonstrated a markedly higher infiltration (30.0 ± 8.2 cells mm^−2^). The inflammatory reaction score was also notably higher in mice with PET implants and in a few with Ag2, though the latter was not significant.

IHC staining (Fig. 9E) targeted α-SMA and CD163 to identify smooth muscle cells and M2 macrophages, respectively. Smooth muscle cells remained structurally intact across all groups, indicating minimal tissue damage from implantation. M2 macrophages, associated with anti-inflammatory activity, were absent in the control group (due to lack of immune activation) and in PET implants despite high leukocyte infiltration, suggesting a predominance of pro-inflammatory M1 polarisation. In contrast, Ag2 coating implants displayed CD163-bearing cells, a favourable sign of inflammatory modulation. Quantitative FI measurements (Fig. 9I) revealed CD163 levels of 141.2 ± 114.7 RFU × 10^3^ in Ag2 samples, significantly higher than 6.4 ± 2.4 RFU × 10^3^ in controls. This effect was attributed to the PEG outer layer of the coating, which helped regulate immune cell responses, encouraging polarisation towards the M2 phenotype and thereby promoting wound healing. Overall, these results confirmed the biocompatibility and strong application potential of PEGylated AgIONPs coatings for biomedical use.

## 4 Conclusion

In this study, a novel surface coating strategy was proposed using modified silver nanoparticles, referred to as AgIONPs/nanostars, in combination with PEG to achieve multifunctional properties, including antithrombogenic and antibacterial effects, as well as on-demand thrombolysis and biofilm disruption via 808-nm laser irradiation. The AgIONPs were successfully synthesised with a spiky morphology, exhibiting a strong NIR absorption profile and a photothermal conversion efficiency of 26.2 %, making them well-suited for 808-nm laser applications due to their effective tissue penetration capacity. The nanoparticles were immobilised onto PET substrates through *in situ* PDA deposition, which also enhanced the photothermal performance of the silver nanostars.

The incorporation of PEG as the outermost coating layer was validated via protein adsorption assays, demonstrating its ability to reduce biofouling, improve haemocompatibility, and enhance cytocompatibility. Antibacterial evaluation confirmed that the coatings effectively inhibited the growth of both Gram-negative *E. coli* and Gram-positive *S. aureus*. Optimised photothermal thrombolysis conditions were identified as 1.5 W cm^−2^ laser intensity, 4 min exposure time, and 3 irradiation cycles. Under these parameters, Ag2 coatings, with an AgIONPs loading of 0.2 mg mL^−1^, achieved maximum temperature elevations below 45 °C, a level considered safe for adjacent tissues.

The photothermal functionality enabled efficient thrombolysis, demonstrated not only in static *in vitro* well plate models but also under dynamic flow conditions. Furthermore, the antibiofilm capability was significant, with most biomass being disrupted after a single irradiation cycle. The coatings exhibited high biocompatibility in cellular assays, *in ovo* CAM testing, and *in vivo* mouse models, with results showing enhanced biocompatibility and reduced immune-mediated inflammatory responses.

Collectively, these findings highlight the AgIONPs–PEG coatings as a novel, advanced photothermal material with dual passive and active functionalities, providing sustained antibiofouling and bactericidal protection, while also offering the capacity to actively counteract thrombosis or biofilm formation on blood-contacting biomedical devices when required.

## CRediT authorship contribution statement

**Cuong Hung Luu:** Writing – review & editing, Writing – original draft, Visualization, Validation, Methodology, Investigation, Conceptualization. **Shehzahdi S. Moonshi:** Methodology, Investigation. **Akriti Nepal:** Methodology, Investigation. **Binura Perera:** Investigation. **Dimple Sajin:** Investigation. **Haotian Cha:** Methodology, Investigation. **Dieu Ngoc Nguyen:** Investigation. **Nam-Trung Nguyen:** Writing – review & editing, Supervision. **Hang Thu Ta:** Writing – review & editing, Supervision, Resources, Project administration, Funding acquisition, Conceptualization.

## Declaration of competing interest

The authors declare the following financial interests/personal relationships which may be considered as potential competing interests: Hang Thu Ta reports financial support was provided by National Health and Medical Research Council. Hang Thu Ta reports financial support was provided by Australian Research Council. If there are other authors, they declare that they have no known competing financial interests or personal relationships that could have appeared to influence the work reported in this paper.

## Data Availability

The data supporting this article have been included in the main manuscript and as part of the Supplementary Information.
