# Supplementary material for: Antithrombotic and antibacterial surface coating based on spiky silver nanoparticles: A counterattack against clotting and biofilm
Source: Mater Today Bio. 2026 Jan 6;37:102762. doi: 10.1016/j.mtbio.2026.102762 (PMC12829136; doi:10.1016/j.mtbio.2026.102762)
Supplement: Multimedia component 1 [file mmc1.docx]

SUPPLEMENTARY INFORMATION

Antithrombotic and antibacterial surface coating based on spiky silver nanoparticles: A counterattack against clotting and biofilm

Cuong Hung Luu ^1^, Shehzahdi S. Moonshi ^1^, Akriti Nepal ^1^, Binura Perera ^1^, Dimple Sajin ^1^, Haotian Cha ^2^, Dieu Ngoc Nguyen ^4^, Nam-Trung Nguyen ^2,3^, Hang Thu Ta ^1,2,*^

^1^ School of Environment and Science, Griffith University, Nathan, QLD 4111, Australia

^2^ Queensland Quantum and Advanced Technologies Research Institute, Griffith University, Nathan, QLD 4111, Australia

^3^ School of Engineering and Built Environment, Griffith University, Nathan, QLD 4111, Australia

^4^ Biomaterials and Nanotechnology Research Group, Faculty of Applied Sciences, Ton Duc Thang University, Ho Chi Minh City, Vietnam

^*^ Corresponding authors:

**Hang Thu Ta**, BEng, MSc, PhD

**Professor**, School of Environment and Science, and Queensland Micro- and Nanotechnology, Griffith University, Nathan Campus, Brisbane QLD 4111, Australia

**Australian Research Council Future Fellow**

**Australian Heart Foundation Future Leader Fellow**

Office: +61 (7) 3735 5384

Email: h.ta@griffith.edu.au

Website: https://hangta.group/

https://experts.griffith.edu.au/27034-hang-ta

Extinction coefficient

To investigate the photo-absorbance characteristics and photothermal performance of AgIONPs, we determined the extinction coefficient of the nanoparticles at various wavelengths and assessed their photothermal conversion efficiency at 808 nm. The extinction coefficient (*ε*, M⁻¹⸱cm⁻¹) was derived according to the Beer–Lambert law as shown in **Equation (1)**, providing insight into the optical behaviour of the system across the examined spectral range.

|  | $\varepsilon=\frac{A}{L\times C_{AgIONPs}}$ |  |
| --- | --- | --- |

Here, *A* (λ) denotes the absorbance at a given wavelength, *L* is the path length (2.8 mm), and *C*_AgIONPs_ (mol⸱L⁻¹) refers to the molar concentration of AgIONPs. The molar concentration, *C*_AgIONPs_, was determined using a calculation method described below, allowing for a quantitative analysis of nanoparticle concentration in solution.

|  | $C_{AgIONPs}=\frac{C_{wt}}{V_{AgIONPs}\times\rho\times N_{A}}$ |  |
| --- | --- | --- |

Where *C*_wt_ (g⸱L⁻¹) denotes the weight concentration, while *N_A_* (6.0221 × 10²³ mol⁻¹) represents Avogadro’s constant. The terms *V*_AgIONPs_ and *ρ* correspond to the average volume and density of the nanoparticles, respectively. For the purpose of this calculation, it was assumed that the AgIONPs adopted an idealised spherical geometry, facilitating estimation of their molar concentration.

The calculated *C*_AgIONPs_ was 3.1 × 10⁻¹² M, taking into account *C*_wt_=0.05 g⸱L⁻¹, *V*_AgIONPs_=2.6 × 10⁻¹⁵ cm³, and *ρ*=10.5 g⸱cm⁻³. The parameters outlined above were referenced and calculated based on the group’s prior research [1]. This calculation was made under the assumption that the nanocomplex consisted predominantly of silver, as confirmed by ICP analysis of Ag and Fe content. The extinction coefficients of AgIONPs at various wavelengths are provided in the table below.

- - - 1. Extinction coefficient of AgIONPs at different wavelengths

| **Wavelength (nm)** | **Absorbance (a.u.)** | ***ε* (M⁻¹⸱cm⁻¹)** |
| --- | --- | --- |
| **680** | 1.8249 | 2.10 × 10¹² |
| **714** | 1.8641 | 2.15 × 10¹² |
| **734** | 1.8963 | 2.18 × 10¹² |
| **780** | 1.9334 | 2.23 × 10¹² |
| **808** | 1.9544 | 2.25 × 10¹² |
| **860** | 1.9572 | 2.25 × 10¹² |
| **924** | 1.9376 | 2.23 × 10¹² |

The results obtained at these wavelengths indicated that AgIONPs exhibited a notably high extinction coefficient. In particular, at 808 nm, the nanoparticles displayed an extinction coefficient of ε=2.23 × 10¹² M⁻¹⸱cm⁻¹, suggesting that AgIONPs were capable of generating an efficient photothermal effect upon exposure to NIR irradiation.

Photothermal conversion efficiency

The photothermal conversion efficiency was determined using the method described by Liu *et al.*, ensuring consistency with established analytical approaches [2]. This calculation provided a quantitative measure of the ability of AgIONPs to convert absorbed NIR energy into heat under the experimental conditions employed.

|  | $\eta=\frac{h\times S\times\left( T_{max}-T_{am} \right)-Q_{dis}}{I\times\left( 1-{10}^{{-A}_{808}} \right)}$ |  |
| --- | --- | --- |

Where *T*_max_ and *T*_am_ represent the maximum temperature under laser irradiation and the ambient temperature, respectively. *Q*_dis_ denotes the heat dissipated by the solvent and container, defined here as 0.004 W⸱K⁻¹. The laser power intensity, *I*, was set at 1.0 W, while *A*₈₀₈ corresponds to the absorbance of AgIONPs at 808 nm, measured at 1.36. The parameter *h* refers to the heat transfer coefficient and *S* to the surface area of the container. Furthermore, the value of *h* × *S* was determined using the equation provided below.

|  | $h\times S=\frac{m\times C}{\tau_{s}}$ |  |
| --- | --- | --- |

Here, *m* denotes the sample mass, measured roughly at 0.1 g, and *C* is the specific heat capacity of water, taken as 4.2 J·g⁻¹·K⁻¹. The parameter *τ*_s_ represents the thermal time constant, which was calculated using **Figure S1** and the equation provided below.

|  | $\theta(t)=\frac{T(t)-T_{am}}{T_{max}-T_{am}}=e^{-\frac{t}{\tau_{s}}}$ |  |
| --- | --- | --- |

Consequently, the photothermal conversion efficiency of AgIONPs was calculated to be approximately 26.2%, reflecting their capacity to effectively convert absorbed NIR energy into heat under the specified experimental conditions.


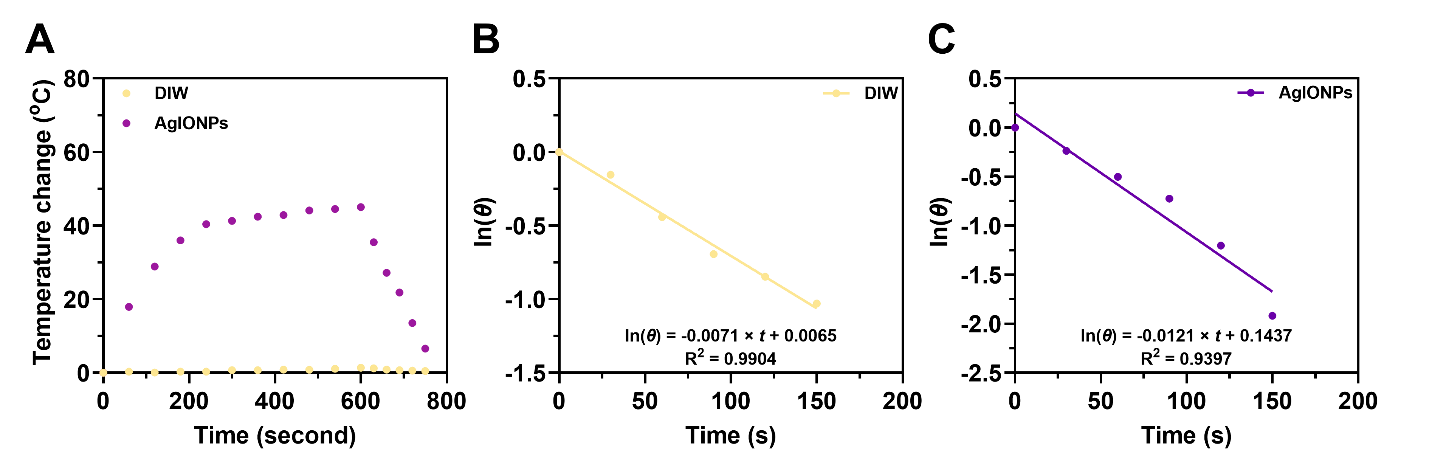


- - 1. Photothermal effect of AgIONPs dispersed in DIW. **(A)** Temperature variation under NIR laser irradiation (808 nm, 1.0 W⸱cm⁻², 600 s laser on, 200 s laser off). Linear plots of time versus ln(*θ*) obtained from the cooling phase of **(B)** DIW and **(C)** AgIONPs samples.
       1. Elemental analysis of Ag and Fe content by ICP-OES for AgIONPs and Ag1–5 samples

| **Samples** | **AgIONPs** | **Ag1** | **Ag2** | **Ag5** |
| --- | --- | --- | --- | --- |
| **Ag (mg⸱L⁻¹)** | 824.2 | 1.6 | 1.9 | 2.1 |
| **Fe (mg⸱L⁻¹)** | 19.0 | < 1.0 | < 1.0 | < 1.0 |


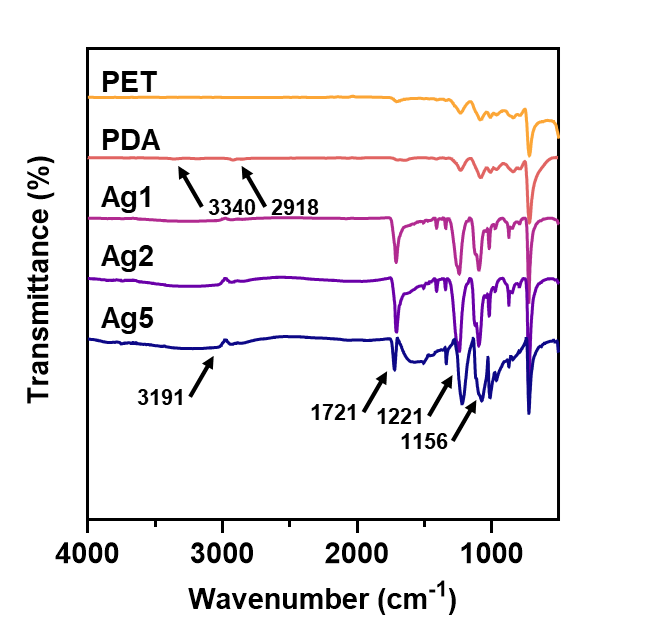


- - 1. FTIR spectra of surface coatings.

Surface engergy analysis

Water contact angle (WCA) measurements for the surface coatings were conducted at ambient temperature using a contact angle system (Attension® Theta Flex, Biolin Scientific AB, Sweden). The assessment was performed employing the sessile drop method, in which 5 µL droplets of distilled water (DIW), diiodomethane (DIM), and dimethylformamide (DMF) were deposited, and their profiles were captured and analysed using the OneAttension software. For each coating sample, no fewer than three independent measurements were taken to determine the contact angle. The surface energy of each sample was subsequently calculated according to Fowkes theory, enabling determination of the polar (*γ*^p^) and dispersive (*γ*^d^) components. In this context, the liquid surface tension and solid surface tension were denoted as *γ*_L_ and *γ*_S_, respectively.

|  | $\gamma_{L}\left( 1+cos \theta\right)=2\sqrt{\gamma_{L}^{p}\cdot\gamma_{S}^{p}}+2\sqrt{\gamma_{L}^{d}\cdot\gamma_{S}^{d}}$ |  |
| --- | --- | --- |

Determination of volumetric flow rate based on physiologically relevant shear rates for microfluidic model

The flow rate employed in the microfluidic model was calculated based on the principles of laminar flow and Newtonian fluid behaviour. The microchannel was designed with precise dimensions, comprising a width of 255 μm, a height of 110 μm, and a length of 20 mm. For ease of estimation, a shear rate calculation approach previously described in the literature was adopted [3, 4]. The experimental setup aimed to replicate a shear rate of approximately 1,000 s⁻¹, which is representative of the physiological conditions typically encountered in most blood vessels. This allowed for the simulation of flow-induced effects on the surface coating under *in vivo*-mimicking conditions. In the case of a rectangular microchannel, the correlation between the shear rate (*γ*) and the volumetric flow rate (*Q*) was approximated using the equation below, where *γ* is expressed in s⁻¹, *Q* in m³·s⁻¹, *w* refers to the channel width in metres, and *h* denotes the channel height in metres.

|  | $\gamma=\frac{6Q}{w\cdot h^{2}}$ |  |
| --- | --- | --- |

The flow rate, initially calculated using the aforementioned equation, was subsequently converted into appropriate units to align with the operational settings of the syringe pump, resulting in a final flow rate of 30.9 μL·min⁻¹.


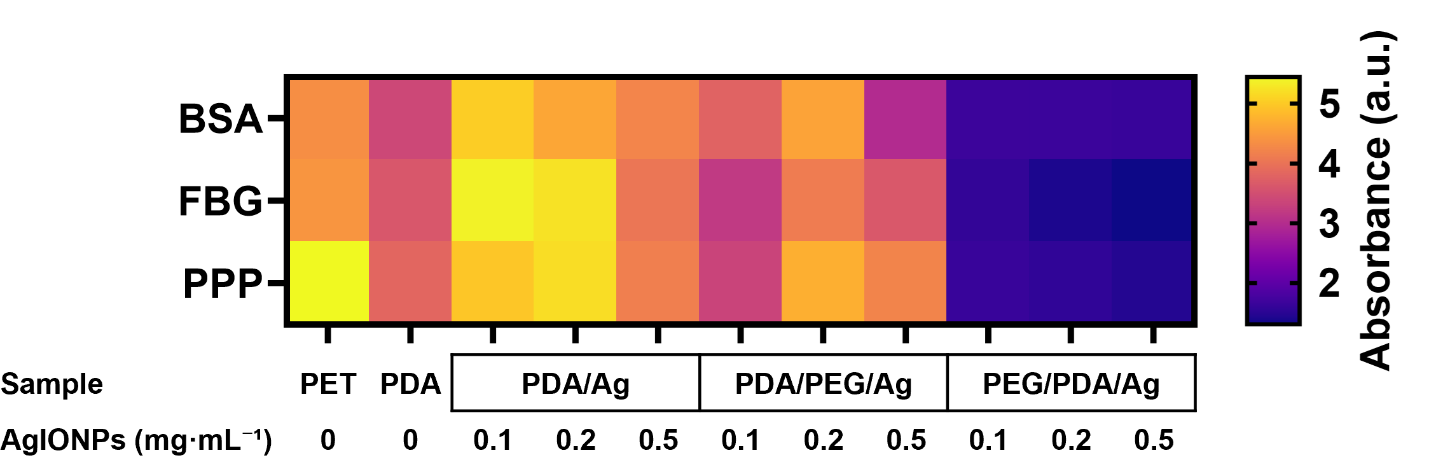


- - 1. Heat map showing preliminary evaluation of surface coating synthesis strategies based on protein adsorption assay.


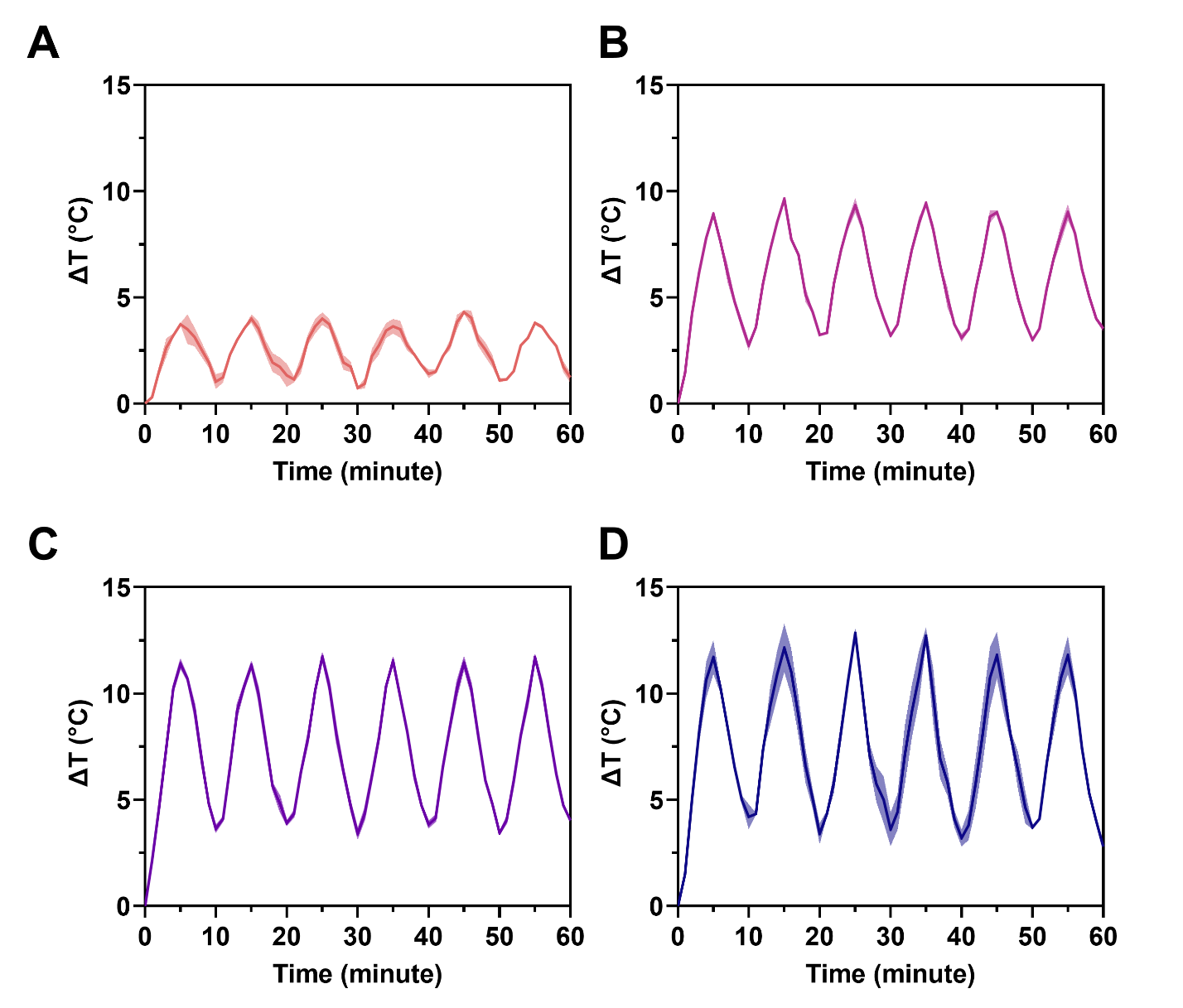


- - 1. Photothermal performance of coated surfaces: **(A)** PDA, **(B)** Ag1, **(C)** Ag2, and **(D)** Ag5 when immersed in DIW. The evaluation was conducted with the laser on for 5 minutes, off for 5 minutes, intensity at 1.5 W·cm⁻², and temperature recorded at 1-minute intervals.


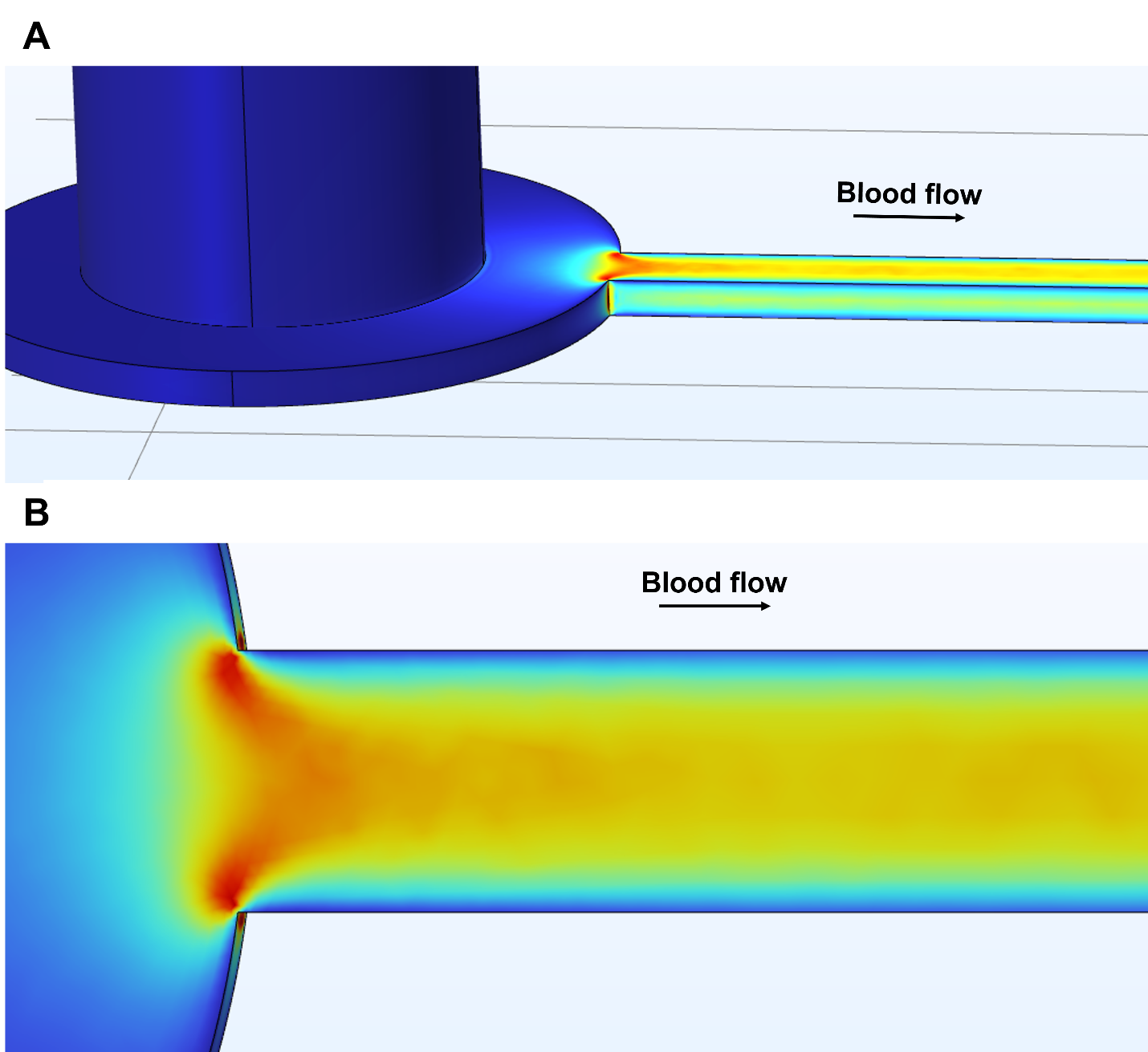


- - 1. Fluid dynamics simulation by COMSOL Multiphysics software showing highly distributed shear stress at the fluid inlet observed in **(A)** side view and **(B)** top view.


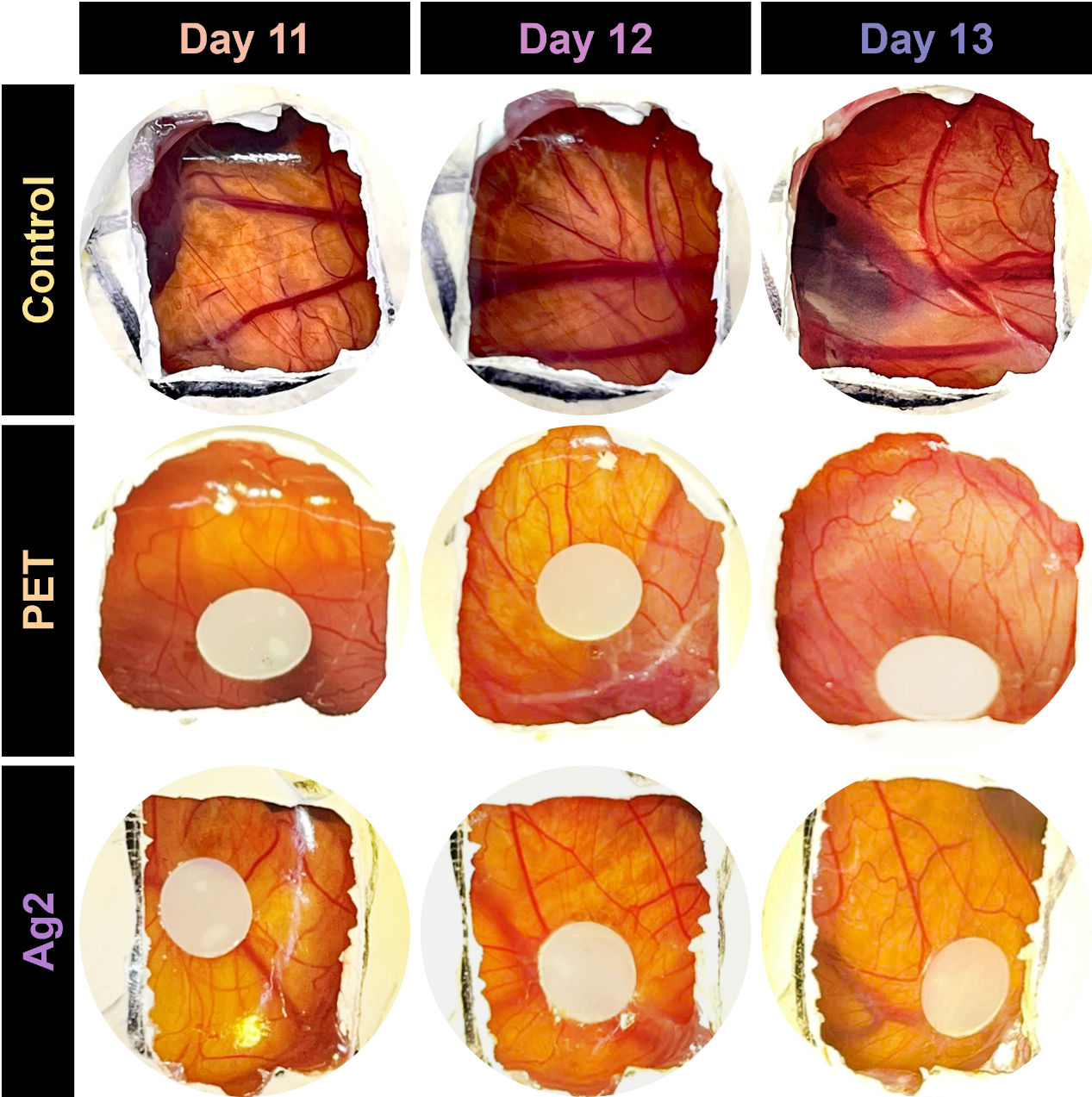


- - 1. Embryonic development captured on days 11, 12, and 13 in conjunction with the implantation of Ag2 coatings.

References

1. Vazquez-Prada, K.X., et al., *A Spiky Silver-Iron Oxide Nanoparticle for Highly Efficient Targeted Photothermal Therapy and Multimodal Imaging of Thrombosis.* Small, 2023. **19**(11): p. 2205744.

2. Liu, X., et al., *Facile synthesis of biocompatible cysteine-coated CuS nanoparticles with high photothermal conversion efficiency for cancer therapy.* Dalton Transactions, 2014. **43**(30): p. 11709-11715.

3. Akther, F., et al., *Atherothrombosis-on-Chip: A Site-Specific Microfluidic Model for Thrombus Formation and Drug Discovery.* Adv. Biol., 2022. **6**(7): p. 2101316.

4. Zhao, Y.C., et al., *Hemodynamic analysis for stenosis microfluidic model of thrombosis with refined computational fluid dynamics simulation.* Sci. Rep., 2021. **11**(1): p. 6875.
